# Systematic review and meta-analysis on the adjunctive use of host immune modulators in non-surgical periodontal treatment in healthy and systemically compromised patients

**DOI:** 10.1038/s41598-021-91506-7

**Published:** 2021-06-09

**Authors:** Stefano Corbella, Elena Calciolari, Alice Alberti, Nikolaos Donos, Luca Francetti

**Affiliations:** 1 Department of Biomedical, Surgical and Dental Sciences, Università degli Studi di Milano, Milan, Italy; 2 IRCCS Istituto Ortopedico Galeazzi, Milan, Italy; 3 Department of Oral Surgery, Institute of Dentistry, I. M. Sechenov First Moscow State Medical University, Moscow, Russia; 4 Centre for Oral Clinical Research and Centre for Oral Immunobiology and Regenerative Medicine, Institute of Dentistry, Barts and The London, School of Medicine and Dentistry, Queen Mary University of London, London, UK; 5 Department of Medicine and Surgery, Centre of Dentistry, University of Parma, Parma, Italy

**Keywords:** Oral diseases, Periodontitis

## Abstract

Considering the central role of inflammation in the pathogenesis of periodontitis, the combination of NSPT with different agents that can modulate the host immune-inflammatory response has been proposed to enhance the outcomes of NSPT. The aim of this paper is to systematically review the literature on the efficacy of systemic host modulators (HMs) as adjuncts to non-surgical periodontal therapy (NSPT) in improving pocket depth (PD) reduction and clinical attachment level (CAL) gain in healthy and systemically compromised patients. RCTs with ≥ 3 months follow-up were independently searched by two reviewers. Meta-analysis was performed when ≥ 3 studies on the same HM were identified. The quality of the evidence was rated according to the GRADE approach to rate the certainty of evidence. 38 articles were included in the qualitative assessment and 27 of them were included in the meta-analysis. There is low/very low evidence that the adjunctive use of sub-antimicrobial dose of doxycicline, melatonin and the combination of omega-3 and low dose aspirin (in type 2 diabetic patients) to NSPT would improve PD and/or CAL. Conflicting evidence is available on the efficacy of probiotics. Future studies controlling for confounding factors, using composite outcomes to define the endpoint of therapy and considering not only the patient- but also as the site-specific effect of systemic HMs are warranted. The dosage, posology and long-term effect of HMs still need to be clarified, also in association to the presence of systemic conditions potentially affecting the response to HMs administration.

## Introduction

Periodontitis is a biofilm-induced chronic inflammatory disease of the tooth-supporting tissues. It has been extensively demonstrated that, while the tooth-associated microbial biofilm is essential to develop the disease, the desctruction of the periodontium is caused by the exaggerated immune-inflammatory host response to the microbial challenge^1^.

Recent advancements coming from independent microbiology studies support a new model in the pathogenesis of periodontitis, namely the polymicrobial synergy and dysbiosis (PSD) model. According to PSD the disease is initiated by a broadly-based dysbiotic and synergistic microbiota, where keystone species (such as *P. gingivalis*) play an important role in triggering the disease^2^. These microorganisms, even at low abundance, are able to engage in a two-way communication with the microbial community inhabitants in order to both impair host immune surveillance and elevate the pathogenicity of the entire microbiota. Inflammation seems to drive the selection and enrichment of the periodontitis-associated microbiota, which is therefore defined as “inflammophilic”^3^, meaning that their presence further sustains the periodontal inflammation.

The goal of periodontitis treatment is to resolve the gingival inflammation and restore periodontal health, ideally with a *restitutio ad integrum* of the damaged tissues and it always starts with the non-surgical periodontal therapy (NSPT). In particular, the first step in therapy aims to guide a behaviour change in patients by motivating them to proper and effective oral hygiene and it also includes risk factors control. This phase should be implemented in all periodontitis patients, irrespective of their disease stage, to facilitate their compliance and it represents the foundation for an optimal treatment response and long-term outcomes. The second step of therapy aims at controlling (reducing/eliminating) the subgingival biofilm and calculus through subgingival manual instrumentation and needs to be followed for all periodontitis patients, irrespective of their disease stage, for all teeth with loss of periodontal support and/or periodontal pocket formation^4^. Although NSPT has proven to be effective in reducing probing pocket depths and improving clinical attachment level^5,6^, its predictability may vary in relation to different factors related to the site (e.g. pocket depth, furcation involvement), the patient (e.g. supragingival plaque control, maintenance care, concomitant systemic diseases, smoking) and the clinician (e.g. effective removal of the biofilm, patient motivation)^7^. Hence, a third step of therapy may be required, which is aimed at treating those sites that did not adequately respond to the second stage of therapy (residual pockets ≥ 4 mm with bleeding on probing and deep pockets ≥ 6 mm) and it may include the repetition of subgingival instrumentation with or without adjunctive therapies and/or different types of periodontal surgeries^4^.

Considering the central role of inflammation in inducing periodontal tissue breakdown and in selecting and sustaining the periodontitis-associated microbiota, the combination of NSPT with different agents that can modulate the host immune-inflammatory response has been proposed to further enhance the outcomes of NSPT, thus possibly reducing the need for subsequent surgeries. A range of host modulating agents that can either block the immune-inflammatory response or promote the natural resolution of the inflammation has been investigated in the past years with heterogeneous results.

The present systematic review aimed to critically evaluate the efficacy of systemic host modulators as adjunctive therapy to NSPT in light of the most recent evidence and to complement the recent review by Donos et al.^8^ by also informing on the short-term effect (3 months) of host modulators and on their use in systemically compromised patients to provide a comprehensive evidence-based guidance for clinicians following the Grading Recommendations Assessment, Development and Evaluation (GRADE) system with the aim of knowing how much confidence we can have in the results of the review.

## Methods

The study protocol was registered in PROSPERO (http://www.crd.york.ac.uk/PROSPERO) with the registration number CRD42018088683 in February 2018, before the beginning of the research.

The protocol is compliant with the Cochrane Handbook^9^ and the results were presented following the instructions of the Preferred Reporting Items for Systematic Review and Meta-analysis (PRISMA) statement^10^.

### PICO question

In human subjects with any form of periodontitis, does the adjunctive use of host-modulator drugs increase the clinical efficacy of non-surgical periodontal therapy (P: humans with periodontitis; I: non-surgical periodontal therapy plus systemically delivered host-modulator drugs; C: non-surgical periodontal therapy alone or combined with placebo; O: clinical outcomes (probing depth (PD) reduction, clinical attachment level (CAL) gain)?

### Search strategy

The following electronic databases were searched for pertinent papers: MEDLINE / PubMed, Scopus, ISI Web of Science, EMBASE and Cochrane Central using a search strategy presented in Appendix 1. Grey literature was searched for pertinent articles interrogating Greylit and OpenGrey. A manual search of the reference lists of the included papers and of the table of contents (since 1990) of Journal of Clinical Periodontology, Journal of Periodontology, Journal of Periodontal Research, Journal of Dentistry, Journal of Dental Research was also performed. Conference abstracts were excluded and only articles in English were considered. The last electronic search was performed on 12th April 2020. A two-stage screening process (titles and abstract first followed by full-text) was performed by two independent reviewers (SC, EC).

### Inclusion criteria

1. Types of studies included: randomized controlled clinical trials with at least 3-month follow-up calculated from the beginning of the treatment protocol
2. Study population: adult (≥ 18 years old) patients affected by periodontitis, either systemically healthy or systemically compromised (e.g. with type 2 diabetes mellitus)
3. Intervention: *Test group*—NSPT protocol (including mechanical treatment using manual curettes and / or ultrasonic devices without the use of antimicrobial agents) combined with the use of a systemic host modulator including but not limited to non-steroidal anti-inflammatory drugs (NSAIDs), bisphosphonates, unsaturated fatty acids, statins, sub-antimicrobial dose of doxycycline, probiotics, micronutrients, melatonin; *Control group*—the same NSPT protocol alone or associated with a placebo
4. Outcomes: *Primary outcome*—reduction in probing depth (PD) and/or clinical attachment loss (CAL) collected at patient level. The primary outcomes can be referred to all the teeth in the mouth or to all the teeth with periodontal pockets (PD > 4 mm); *Secondary outcomes*—changes in plaque scores, bleeding/inflammation scores, adverse events and patient-reported outcome measures (PROMs)

The studies had to provide a complete description of the host modulator prescribed, meaning the presentation of the active substance, concentration and dosage in order to be considered for this review.

Studies with a split-mouth design, and studies presenting data only on a sample of the teeth were excluded. Studies reporting duplicated data (the same data published elsewhere) were excluded.

Cohen’s kappa served to evaluate the concordance in the selection of the two authors.

Disagreements in article selection processes were solved by consulting a third reviewer (AA) whose opinion was considered diriment.

### Data extraction

Three authors (SC, EC, AA) independently extracted the following data from the included studies: author names, year of publication, country of recruitment and treatment, sample characteristics (size, ethnicity, gender distribution, smoking status, mean age or age groups), definition / diagnostic criteria of periodontal disease, clinical data before and after the treatment (mean periodontal probing depth (PD), mean clinical attachment level (CAL), gingival bleeding indexes (Gingival Bleeding index^11^, Gingival index-GI—^12^, percentage of bleeding sites—BOP -), plaque indexes (Plaque index^13^, Turesky-modified plaque index^14^, proportion of sites with visible plaque) or difference between baseline and follow-up values. The occurrence of adverse events and all patients’ reported outcomes (PROMs) were recorded.

An attempt was made to contact by email the authors of the papers providing insufficient information.

### Risk of bias evaluation

The risk of bias evaluation and quality assessment of all included papers was performed by two reviewers (SC, AA) and any disagreement was resolved by discussion. The criteria considered for risk of bias evaluation were extrapolated from the *Cochrane Handbook for Systematic Reviews*^9^ (Cochrane risk-of-bias tool for randomized trials) and they included:

- Bias arising from the randomization process
- Bias due to deviations from intended interventions
- Bias due to missing outcome data
- Bias in measurement of the outcome
- Bias in selection of the reported result

The overall risk-of-bias judgement was *high risk* if the level of risk of bias was judged to be high for at least one domain or if the trial was judged to have some concerns for multiple domains (three). If the trial was judged to have some concerns for less than three domains the overall risk of bias was *“some concerns”*, while the study was judged to have *low risk* of bias if all domains were judged to have low risk.

The funding bias was estimated by evaluating if authors disclosed their potential sources of competing conflict of interest and the source of funding for the studies they carried on (if any).

### Meta-analysis, assessment of heterogeneity and assessment of reporting biases

For quantitative analysis, studies were grouped according to the HM employed, follow-up time and, whenever possible, according to the initial PD. Meta-analysis was performed using the software RevMan (Review Manager Version 5.3, 2014; The Nordic Cochrane Center, The Cochrane Collaboration, Copenhagen, Denmark) if at least three papers were available for each comparison. A sub-analysis was performed when three or more studies were available for one specific active principle within the same category of HMs (e.g. ibuprofen among FANS or one specific probiotic).

For each presented outcome, the difference between baseline and follow-up values were extracted (with specific error measure such as standard deviation (SD) or standard error (SE) or variance). When such parameter was not presented, it was computed as the difference between baseline and follow-up values. In these cases, following the instructions of the Cochrane Handbook for Systematic Reviews when SDs of changes values were not presented and they were not provided by authors after contacting them by email, they were computed as follows: (1) if similar studies were present (similar treatment, similar population, similar sample size), SD was imputed taking the value of the other study; (2) when *P* value is presented SD was computed by using T tables for retrieving SEs; (3) when *P* value is presented as a limit (e.g. < 0.05) a conservative value of *P* (e.g. 0.05 in case of < 0.05) was considered for computing SE as described before; (4) if *P* value was not present SDs of change values was imputed by using the following formula^9,15,16^:

$$SDcv = \sqrt {SD\;baseline^{2} + SD\;final^{2} - \left( {2*CORR*SD\;baseline*SD\;final} \right)}$$

being CORR the correlation coefficient, that could be imputed from similar studies if present, or it was assumed conservatively to be 0.2. For each measure, pooled estimate of 95% CI was calculated.

In the meta-analysis effect size was computed through the weighted mean method and results were combined using the DerSimonian and Laird’s random-effect model^17^, assuming heterogeneity among studies.

Cochran’s test served to measure the consistency of the results, considering it significant if *P* < 0.1. I^2^ statistics was applied to measure heterogeneity (total variation across studies that was due to heterogeneity rather than to chance). If I^2^ was less than 40% the heterogeneity was negligible, if it was from 40 to 60% it signified a moderate heterogeneity, if 60% to 90% it signified a substantial heterogeneity while it showed a considerable heterogeneity if it was from 75 to 100%^18^.

Small study effects, as proxy for publication bias, were assessed by testing for funnel plot asymmetry and by calculating Egger´s bias, as described in the Cochrane Handbook (Higgins and Green 2011).

### Quality of evidence assessment

The quality of the available evidence was assessed for each comparison and for each outcome included in the meta-analysis through the Grading of Recommendations, Assessment, Development and Evaluations (GRADE) approach. GRADE provides a system for rating quality of evidence and strength of recommendations that is explicit, comprehensive, transparent, and pragmatic^19^. More specifically, GRADE indicates four grades of evidence (high quality, moderate, low, and very low) and the strength of recommendation is qualified as strong, weak, or conditional to an intervention (pro or con) for each specific comparison and outcome. The GRADE approach implies the consideration of the risk of bias of the studies, of inconsistency (heterogeneity), of indirectness of evidence, of imprecision of the effect estimates and of risk for publication bias.

## Results

**Figure 1:**
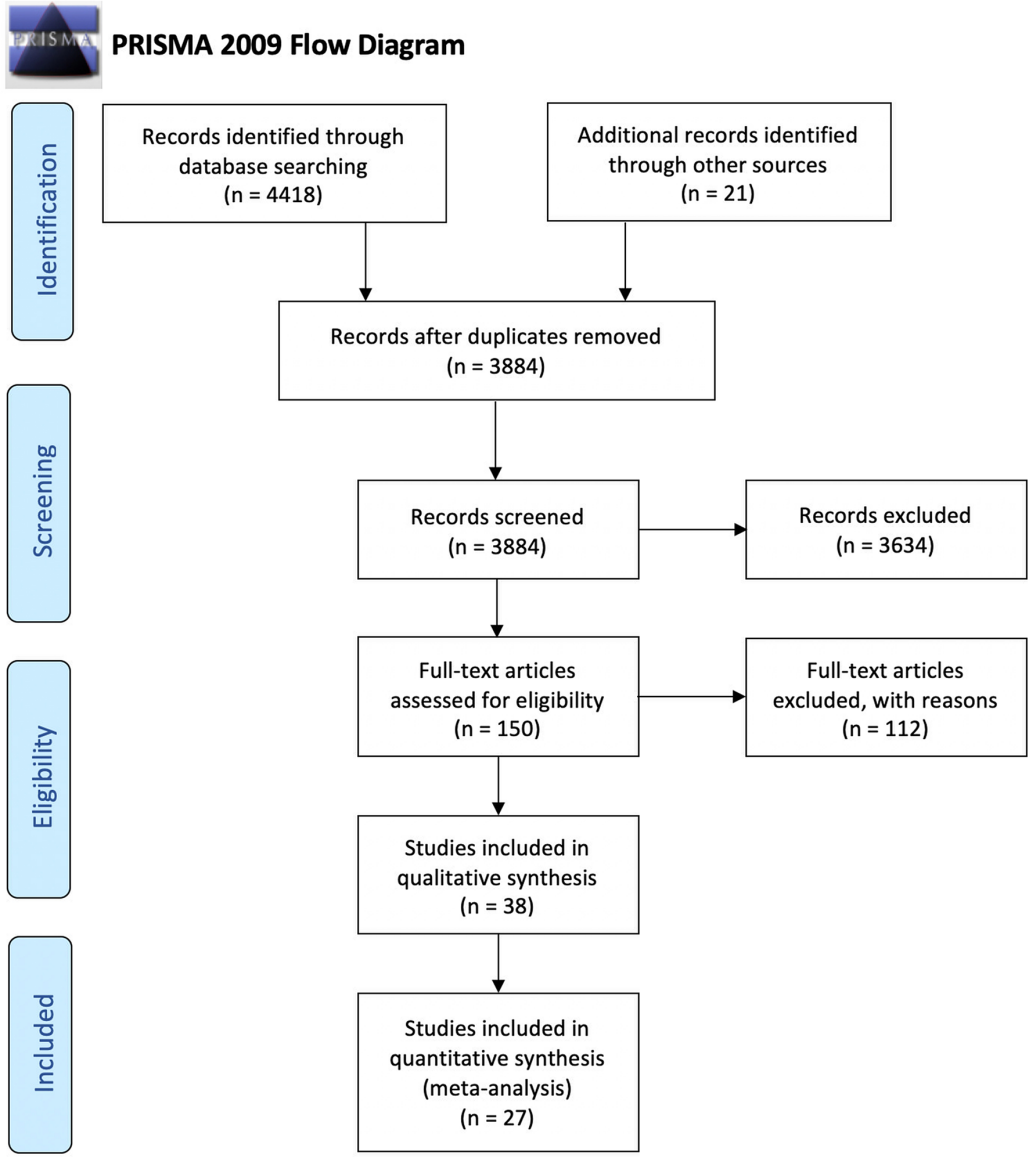
Study flow diagram.

The article selection process is summarized in Fig. 1. The electronic and manual search retrieved a total of 3884 papers, whose titles and abstract were assessed for eligibility. A total of 150 full text articles were checked for inclusion. Of those, a total of 38 articles were included in the qualitative assessment and 27 of them were included in the meta-analysis. Kappa of agreement during the selection process was > 0.9 for titles and abstracts, as well as for full texts. Reasons for exclusions of studies at the full-text stage are reported in Appendix 2.

The studies included were published from 2004 to 2020 and they were carried on in various countries, 25 of them in University settings, two in private practices, one in University and private setting and the others did not provide any information about the setting.

The summary of the characteristics of the included studies are presented in Table 1. Briefly, eight out of 38 studies did not have a placebo control group (17.9%), and five had two test groups. With regard to the characteristics of the population, one study examined a sample made only of postmenopausal women^20^, one was on elderly people (≥ 65 years old)^21^, four studies on former or current smokers^22–25^, and one study included only smokeless tobacco users^26^. For systemically compromised patients five studies included only subjects with treated type 2 diabetes^27–30^, and the other studies were on systemically healthy subjects. The maximum reported follow-up was 12 months.

**Table 1:** Main characteristics of the included studies. Abbreviations and references used in this table are reported in Appendix 6.

Authors and year	Study characteristics	N° subjects	Sex	Age mean ± SD (age range) years	Systemic conditions	Country/Ethnicity	Periodontal disease	Outcomes	Type of probe and N sites/tooth evaluated	NSPT details	Follow-up	Negative control group (G0)	Test group 1 (G1)	Test group 2 (G2)
Rocha et al. (2004)	RCT	40	40F	55–65; G0: 58.0 ± 2.8; G1: 57.8 ± 2.9	Postmenopausal women; non-diabetic subjects	NS	At least 3 teeth with PD ≥ 3 mm, GI of 2 or 3, PI 2 or 3, gingival recession, and a minimum of 15 teeth	PD, REC, CAL, %mobility, %BOP, %PI, tooth loss, radiographic bone loss, calcaneus BMD, serum NTx, serum BSAP, blood levels of FSH, LH, gonadotropins and steroid hormones	Michigan probe type O; 6 sites per tooth	SRP in 4 sessions	6 mo from beginning of SRP + HM administration	NSPT + placebo (ND)	NSPT + Alendronate 10 mg once a day for 6 mo	–
Lee et al. (2004)	RCT, double-blind, parallel	66 (41 analyzed)	NS	NS	Systemically healthy; not smoking over the past year	NS	CP: at least 4 teeth with PD of 5–9 mm in 3 or 4 qualifying quadrants	CAL, PD, GCF levels, MMP-8 and -13 levels, analysis of periodontal microflora	automated probe (Florida Disc Probe, Florida Probe Co., Gainesville, FL); 6 sites per tooth	SRP (no futrther details)	1, 3, 6, 9 mo	NSPT + placebo (ND)	NSPT + Doxycycline hyclate 20 mg for 9 mo	–
Preshaw et al. (2004)	RCT, double-blind, multicenter	209	G0: 58 M, 44F; G1: 67 M, 40F	G0: 48 (34–75); G1: 48 (35–75)	Systemically healthy; 122 ex or current smokers	G0: 70 White, 19 Black, 6 Asian, 7 Hispanic; G1: 76 White, 17 Black, 5 Asian, 9 Hispanic	CAL and PD between 5 and 9 mm with BOP in 2 sites in each of 2 quadrants	CAL, PD, %BOP, adverse events	UNC-15 probe, 6 sites per tooth	SRP performed both by hand and ultrasonic instrumentation	3, 6, 9 mo	NSPT + placebo (ND)	NSPT + doxycycline 20 mg twice a day for 9 mo	–
Mohammad et al. (2005)	RCT, double-blind	24	3 M, 21F	G0: 83 (77–90); G1: 81 (72–93)	65 + years; not smoking; systemically healthy	White Caucasian	Moderate-severe CP: CAL 5–9 mm, PD 4–9 mm and BOP in at least 2 non-adjacent periodontal sites	PD, CAL, %BOP	UNC-15 probe, 6 sites per tooth	SRP (no futrther details)	3, 6, 9 mo	NSPT + placebo (ND)	NSPT + doxycycline hyclate 20 mg twice a day for 9 mo	–
Gorska and Nedzi-Gora (2006)	RCT	66	G0: 15 M, 18F; G1: 15 M, 18F	G0: 44 (23–63); G1: 43 (20–56)	Systemically healthy	NS	CP (Armitage 1999)	PD, CAL, %BOP, %BI, %PI (O'Leary et al. 1972), MMP-8, MMP-9, and TIMP-1 concentrations in saliva and peripheral blood	diagnostic Florida Probe (FP32); 6 sites per tooh	SRP performed by a single operator	3 mo	NSPT alone	NSTP + Doxycycline 20 mg twice a day for 3 mo	–
Emingil et al. (2006)	RCT, double-blind	65 (46 analyzed)	46 M, 19F	(34–6); G0: 47.70 ± 7.59 (35–61); G1: 46.11 ± 6.37 (34–59)	Not heavy smokers (< 5 cigarettes/day)	NS	At least 8 sites with PD ≥ 5 mm and CAL ≥ 4 mm, and radiographic evidence of moderate to advanced CP (Armitage 1999)	PD, CAL, GI (Löe and Silness 1963), PI (Turesky et al. 1970), GCF t-PA total amount	Williams probe; 6 sites per tooth	SRP in 4–6 sessions, performed by a single operator	3, 6, 9, 12 mo from beginning of SRP + HM administration	NSPT + placebo (capsules containing starch)	NSPT + Doxycycline 20 mg twice a day for 3 mo	–
Needleman et al. (2007)	RCT, triple-blind, parallel	34 (4 dropouts, 34 analyzed in ITT analysis)	NS	32–58 (at baseline), 32–50 (analyzed)	Current smoker (10 + cigarettes per day for at least 1 year)	NS	CP: at least two teeth with PD ≥ 6 mm and at least 2 quadrants (excluding third molars) with bone loss ≥ 30%	CAL, PD, REC (only sites with initial PD ≥ 5 mm); %BOP, %PI, GCF samples	UNC-15 probe, 6 sites per tooth	SRP + OHI in 4 sessions, performed by a single experienced periodontist, both by hand and ultrasonic instrumentation	1, 3, 6 mo after SRP completion	NSPT + placebo (identical to test group except for doxycycline)	NSPT + 20 mg doxycycline twice a day for 3 mo	–
Preshaw et al. (2008)	RCT, double-blind, multicenter	266 (227 analyzed)	G0: 62 M, 71F; G1: 58 M, 75F	G0: 49.9 ± 11.0 (23–82); G1: 48.5 ± 11.4 (24–81) (of 266)	166 ex or current smokers	G1: 87 White, 28 Black, 8 Asian, 10 other G0: 100 White, 21 Black, 7 Asian, 5 other (of 266)	At least 4 periodontal sites in each of 2 quadrants, at least 2 affected teeth per quadrant, all 8 qualifying sites with PD ≥ 5 mm, CAL ≥ 5 mm, and BOP score ≥ 1, at least 2 sites with bleeding scores ≥ 2	PD, CAL, BOP% (Polson et al. 1995), adverse events, microbiological assessment of subgingival samples	UNC-15 probe, 6 sites per tooth	SRP completed within 24 h, performed both by hand and ultrasonic instrumentation	3, 6, 9 mo	NSPT + placebo (ND)	NSPT + doxycycline 40 mg once a day for 9 mo	–
Alec Yen et al. (2008)	RCT, double-blind	131 (101, 85, 74 and 65 at 3, 6, 9 and 12 mo	54 M, 47F (at 3 mo)	48.6 ± 9.94; G0: 47.3 ± 9.2; G1: 49.6 ± 10.5 (at 3 mo)	Systemically healthy	64 White, 24 Black, 4 Hispanic, 7 Asian, 2 other (at 3 mo)	At least 4 teeth with PD > 4 mm and CAL > 2 mm, and at least 3 interproximal areas with radiographic bone loss	PD, CAL, %BOP, PI (O'Leary et al. 1972), mobility, sites with CAL gain ≥ 2 mm	UNC-15 probe, 6 sites per tooth	SRP in 2 sessions within 1–2 weeks	3, 6, 9, 12 mo	NSPT + placebo (ND)	NSPT + Celecoxib 200 mg once a day for 6 mo	–
Graziani et al. (2009)	RCT, single-blind, parallel, open-label	60 (9 dropouts; 60 analysed in ITT analysis)	21 M, 39F	G0: 42.2 (95% CI 38.7–45.7); G1: 44.7 (95%CI 42.2–47.3)	Systemically healthy	NS	Generalized advanced CP (Armitage 1999)	PD, CAL, % of pockets of different depth, %BOP, %PI, FMPS%, FMBS%	UNC-15 probe, 6 sites per tooth	SRP in 4 sessions within 2 weeks, performed by a single experienced certified therapist both by hand and ultrasonic instrumentation (piezoelectric instrument with fine tips)	3, 6 mo	NSPT alone	NSPT + neridronate 12.5 mg once a week for 12 weeks	–
Abou Sulaiman and Shehadeh (2010)	RCT	30	9 M, 21F; G0: 6 M, 9F; G1: 3 M, 12F	41 (23–65); G0: 42 (30–60); G1: 40 (23–65)	Systemically healthy; not smoking	patients of Syrian descent	CP: at least 2 non-adjacent sites per quadrant that are not first molar or incisor, with PD ≥ 5 mm + bleeding on gentle probing + radiographic bone loss ≥ 30% of the root length (Armitage 1999)	PD, CAL, %BOP, PI (Silness and Löe 1964), GI (Löe and Silness 1963), Plasma TAOC levels	PCP-UNC15 probe; 4 sites per tooth	SRP completed within 48 h, performed by a single periodontist, by hand instrumentation	1, 3 mo	NSPT alone	NSPT + Vitamin C 2000 mg a day for 4 weeks	–
Gilowski et al. (2012)	RCT, double-blind, parallel	34	16 M, 18F	G0: 56.0 ± 9.0; G1: 57.6 ± 8.0	T2DM (at least 6 mo before the study)	NS	Severe or moderate, localized or generalized CP: at least 4 non-adjacent sites with PD ≥ 4 mm	PD, CAL, %BOP, API (Lange et al. 1977), GCF MMP-8 levels	Williams probe; 6 sites per tooth	SRP in 1 session, performed by a single operator both by hand and ultrasonic instrumentation	3 mo	NSPT + placebo (saccharum lactis)	NSPT + doxycycline 20 mg twice a day for 3 mo	–
Chapple et al. (2012)	RCT, double-blind,	61 (60 at 3 mo, 54 at 6 and 9 mo)	21 M, 39F	G0: 47.9 ± 6.6; G1: 48.3 ± 8.4; G2: 48.1 ± 7.4	Systemically healthy; not smoking	NS	At least 2 sites per quadrant with PD or interproximal CAL loss > 6 mm and radiographic bone loss more than 1/3 of the root length	PD, REC, %BOP, MGI (Lobene et al. 1986), modified Quigley-Hein index (Lobene et al. 1982), GCF, blood sample	UNC CP-15 markings—0.2 N force probe; 6 sites per tooth	SRP in 4 sessions within 1 mo, performed by a single experienced certified therapist	3, 6, 9 mo from beginning of SRP	NSPT + placebo (microcrystalline cellulose)	NSPT + FV (declared totals per daily dose: b-carotene 7.5 mg, vitamin E 46 mg, vitamin C 200 mg, folic acid 400 lg) for 8 mo	NSPT + FVB (declared totals per daily dose: b-carotene 7.5 mg, vitamin E 66 mg, vitamin C 222 mg, folic acid 640 lg) for 8 mo
Teughels et al. (2013)	RCT, double-blind, parallel	30	G0: 8 M, 7F; G1: 7 M, 8F	G0: 45.73 ± 6.24; G1: 46.60 ± 4.47	Systemically healthy; not smoking over the past year	NS	Moderate to severe generalized CP (Van der Velden 2005)	PD, REC, %BOP, GI (Löe and Silness 1963), PI (Silness and Löe 1964), microbiological parameters, need for surgery (%sites, %teeth, number of patients)	UNC probe; NS sites per tooth	Full-mouth one-stage disinfection approach (Quirynen et al. 2006): SRP performed on two consecutive days, by a single periodontist both by hand and ultrasonic instrumentation under 0.12% CHX irrigation	3, 6, 9 mo	NSPT + placebo (ND)	NSPT + Probiotic (*L. reuteri* 1E8 CFU) twice a day for 12 weeks	–
Parvu et al. (2013)	RCT, double-blind	174	G0: 43 M, 44F; G1: 42 M, 45F	(30–60); G0: 43.12; G1: 41.5	No uncontrolled systemic diseases; not smoking	NS	moderate to advanced CP: at least 2 sites with PD ≥ 5 mm and BOP + ; CAL ≥ 5-mm; and radiographic evidence of bone loss	PD, CAL, BOP	6 aspects per tooth; standard manual periodontal probe (DB764R, Aesculap AG, Tuttlingen, Germany)	SRP in 4 sessions at 1-week intervals, performed by a single experienced periodontist both by hand and ultrasonic instrumentation	3 mo from beginning of SRP + HM administration	NSPT + placebo (cornstarch)	NSPT + doxycycline 20 mg twice a day for 3 mo	–
Deore et al. (2014)	RCT, double-blind, parallel	60 (58 analysed)	NS (no statistically significant differences reported between groups)	G0: 44.47 ± 5.20; G1: 45.40 ± 40.90;	Systemically healthy; not smoking	NS	Moderate CP: at least 2 interproximal sites with CAL ≥ 4 mm on different teeth or PD ≥ 5 mm on different teeth); Severe CP: at least 2 interproximal sites with CAL ≥ 6 mm on different teeth and at least 1 interproximal site with PD ≥ 5 mm (Page and Eke 2007)	PD, CAL, SBI, GI, OHIS, PI, serum CRP levels	UNC-15 probe, 4 sites per tooth	SRP in 2 sessions within 2 weeks, performed by a single periodontist, both by hand and ultrasonic instrumentation	1.5, 3 mo	NSPT + placebo (capsule containing 300 mg of liquid paraffin)	NSPT + omega-3 PUFAs 300 mg once a day for 2 weeks	–
Singh et al. (2014)	RCT, parallel	38	8 M, 30F	37.50 (17–58)	Systemically healthy; not smoking	NS	CP: at least 2 interproximal sites with CAL ≥ 4 mm, or ≥ 2 interproximal sites with PD ≥ 5 mm, not on the same tooth (Page and Eke 2007)	PD, CAL, %BOP, PI (Silness and Löe 1964), GI (Löe and Silness 1963), serum and salivary SOD activity	NS probe; 6 sites per tooth	SRP performed both by hand and ultrasonic instrumentation	3 mo	NSPT alone	NSPT + Vitamin E 200 mg (300 IU) every other day for 3 mo	–
Laleman et al. (2015)	RCT, double-blind, parallel	48	G0: 14 M, 10F; G1: 12 M, 12F	G0: 47 ± 5 (39–58); G1: 46 ± 5 (37–54)	Systemically healthy; not smoking	NS	Moderate to severe CP (Van der Velden 2005)	PD, REC, %BOP, RAL, PI (Silness and Löe 1964), GI (Löe and Silness 1963), microbiological parameters	UNC probe; 6 sites per tooth	Full-mouth one-stage disinfection approach (Quirynen et al. 2006): SRP performed on two consecutive days, by a single periodontist both by hand and ultrasonic instrumentation under 0.12% CHX irrigation	3, 6 mo	NSPT + placebo (identical to test group except for the probiotic)	NSPT + probiotic (*S. oralis* KJ3, *S. uberis* KJ2 and *S. rattus* JH145, at least 10^8^ CFU of each strain per tablet) twice a day for 3 mo	–
Tekce et al. (2015)	RCT, double-blind, parallel	40	G0: 10 M, 10F; G1: 8 M, 12F	(35–50); G0: 41.40 ± 8.86; G1: 43 ± 5.01	Systemically healthy; not smoking	NS	CP: radiographic horizontal bone loss; at least 2 teeth with one approximal site each with a PD of 5–7 mm and GI ≥ 2 in each quadrant (Armitage 1999)	PD, RAL, %BOP, PI (Silness and Löe 1964), GI (Löe and Silness 1963), microbiological parameters	PCP-UNC 15 probe; NS sites per tooth	SRP in 2 sessions at 1-week intervals, performed both by hand and ultrasonic instrumentation	3 weeks, 3, 6, 12 mo	NSPT + placebo (ND)	NSPT + Probiotic (*L. reuteri*) twice a day for 3 weeks	–
Ince et al. (2015)	RCT, double-blind	30	17 M, 13F; G0: 8 M, 7F; G1: 9 M, 6F	(35–50); G0: 42.20 ± 2.78; G1: 41 ± 3.17	Systemically healthy; not smoking	NS	CP (Armitage 1999)	PD, RAL, %BOP, PI (Silness and Löe 1964), GI (Löe and Silness 1963), MMP-8, TIMP-1	PCP-UNC 15 probe; 6 sites per tooth	SRP in 2 sessions within 1 week, performed both by hand and ultrasonic instrumentation	3 weeks, 3, 6, 12 mo	NSPT + placebo (ND)	NSPT + probiotic (*L. reuteri*) twice a day for 3 weeks	–
Elwakeel and Hazaa (2015)	RCT, double-blind, parallel	40	20 M, 20F	40.05 ± 9 (24–58)	T2DM; not smoking	NS	Moderate to severe CP: ≥ 14 natural teeth, at least 5 teeth with PD ≥ 5 mm and CAL ≥ 4 mm (AAP 2000)	PD, CAL, GI (Löe and Silness 1963), PI (Silness and Löe 1964), GCF levels of IL-1β and MCP-3	Michigan 0 probe with Williams markings; NS number of sites per tooth	SRP in 2 sessions within 2 weeks, performed by a single periodontist, both by hand and ultrasonic instrumentation	3, 6 mo	NSPT + placebo (placebo for aspirin: lactose tablet; placebo for × 3 PUFAs: coconut oil)	NSPT + omega-3 1 g 3 times a day + 75 mg aspirin once a day, for 6 mo	–
Morales et al. (2016)	RCT, double-blind, parallel	28	14 M, 14F; G0: 7 M, 7F; G1: 7 M, 7F	Overall: 49.8 (35–68); G1: 52.7 ± 7.3	Systemically healthy; test group: 4 smokers; control group: 2 smokers	NS	CP: at least 5 teeth with PD ≥ 5 mm and CAL ≥ 3 mm, 20% BOP, and extensive radiographic bone loss (Pozo et al. 2005)	PD, CAL, %PI, %BOP	UNC-15 probe, 6 sites per tooth	SRP in 4–6 sessions at 1-week intervals, performed by two operators both by hand and ultrasonic instrumentation	3, 6, 9, 12 mo after SRP completion	NSPT + placebo (ND)	NSPT + probiotic (*L. rhamnosus* SP1, 2 × 10^7^ CFU/day) once a day for 3 mo	–
Alyousef et al. (2017)	RCT, double-blind, parallel	65	NS (no statistically significant differences between groups)	G0: 39.4 ± 21.6; G1: 34 ± 25.6	Systemically healthy	NS	Moderate CP: at least 2 interproximal sites with CAL ≥ 4 mm or PD ≥ 5 mm); Severe CP: at least 2 interproximal sites with CAL ≥ 6 mm and at least 1 interproximal site with PD ≥ 5 mm	PD, CAL, GI, SBI, PI, OHIS, serum CRP levels, NOS activity, cytokine activity	UNC-15; 4 sites per tooth	SRP performed by a periodontist, either by hand or ultrasonic instrumentation + OHI	1, 2, 3 mo	NSPT + placebo (ND)	NSPT + Incyclinide 20 mg twice a day for 3 weeks	–
Umrania et al. (2017)	RCT, examiner-masked, parallel	40	G0: 13 M, 7F; G1: 12 M, 8F	G0: 43.5 ± 5.8; G1: 44 ± 6.44	Not smoking	NS	At least 30% of the sites with CAL ≥ 5 mm (Flemmig 1999)	PD, CAL PI (Silness and Löe 1964), GI (Löe and Silness 1963), GBI (Ainamo & Bay 1975), salivary levels of IL-1β	NS	SRP (no futrther details)	1, 3 mo	NSPT alone	NSPT + 700 mg fish oil (EPA 180 mg / DHA 120 mg) once a day for 3 mo	–
Chitsazi et al. (2017)	RCT	60	29 M, 31F	41 (23‒65)	Not smoking	Iranians	Moderate-to-severe CP: at least 3 sites with PD of 5–7 mm	PD, CAL, GI	UNC-15; 4 sites per tooth	SRP performed by a single periodontist both by hand and ultrasonic instrumentation	3, 6 mo	NSPT alone	NSPT + Melatonin 2 mg a day for 4 weeks	NSPT + Melatonin 2 mg a day for 4 weeks + Vitamin C 60 mg (females) or 75 mg (males) a day for 4 weeks
Keskiner et al. (2017)	RCT, double-blind, parallel	30	G0: 8 M, 7F; G1: 8 M, 7F	G0: 42.54 ± 5.82; G1: 40.87 ± 9.7	Systemically healthy; not smoking	NS	CP: at least 9 posterior teeth (not including third molars and teeth with bridges and crowns) with PD of 5–7 mm and 3 teeth with PD ≥ 6 mm	PD, CAL, PI (Silness and Löe 1964), GI (Löe and Silness 1963), %BOP, salivary levels of TNF-α and SOD	Williams probe; 6 sites per tooth	SRP performed by a single experienced operator	1, 3, 6 mo	NSPT + placebo (identical to test group except for the fish oil)	NSPT + omega-3 PUFAs (EPA 6.25 mg + DHA 19.19 mg)	–
El-Sharkawy et al. (2019)	RCT, double-blind, parallel	80 (74 analyzed)	G0: 20 M, 16F; G1: 21 M, 17F	G0: 46.7 ± 8.3; G1: 45.6 ± 7.1	Subjects with primary insomnia; not smoking	Saudi Arabia	Generalized CP: radiographic bone loss and presence of PD ≥ 5 mm and at least 3 sites in each quadrant with CAL ≥ 4 mm (Armitage 1999)	PD, CAL, GI (Löe and Silness 1963), %BOP, PI (Silness & Löe 1964), salivary TNF-α levels, AIS scores	NS	SRP in 2 sessions, performed by a single experienced periodontist both by hand and ultrasonic instrumentation	3, 6 mo	NSPT + placebo (ND)	NSPT + melatonin 10 mg once a day for 2 mo	–
Invernici et al. (2018)	RCT, double-blind	41	NS (no statistically significant differences reported between groups)	NS (no statistically significant differences reported between groups)	Systemically healthy; not smoking	NS	Generalized CP (Armitage 1999)	PD, CAL, REC, %PI, GI, %BOP, number of moderate and deep pockets, microbiological parameters	PCPUNC156 probe; 6 sites per tooth	SRP completed within 24 h, performed by a single periodontist both by hand and ultrasonic instrumentation	1, 3 mo	NSPT + placebo (ND)	NSPT + Probiotic (*B. lactis* HN019, 109 CFUs per lozenge) twice a day for 30 days	–
Morales et al. (2018)	RCT, double-blind, parallel	47	G0: 8 M, 7F; G1: 8 M, 8F; G2: 10 M, 6F	G0: 52.8 ± 7.5; G1: 46.5 ± 9.3; G2: 49.0 ± 7.9	Systemically healthy; 16 smokers (7 in probiotic group, 3 in antibiotic group, 6 in placebo group)	NS	CP: at least 5 teeth with PD ≥ 4 mm and CAL ≥ 1 mm, 20% BOP, and extensive radiographic bone loss (Van der Velden 2005)	PD, CAL, %BOP, %PI, microbiological analysis of subgingival plaque samples	UNC-15 probe, 6 sites per tooth	SRP in 4–6 sessions at 1-week intervals, performed by two operators, both by hand and ultrasonic instrumentation	3, 6, 9 mo after SRP completion	NSPT + placebo (ND)	NSPT + probiotic (*L. rhamnosus* SP1, 2 × 10^7^ CFU/ day) once a day for 3 mo	NSPT + antibiotic (Azithromycin 500 mg) once a day for 5 days
Surapaneni et al. (2018)	RCT, single-blind	40	18 M, 22F	(35–60)	Recently diagnosed T2DM, taking metformin 500 mg/day	Indian	CP: at least 4 teeth with PD ≥ 5 mm, CAL ≥ 4 mm and BOP + (Machtei et al. 1992)	PD, CAL, GI	Williams probe; 6 sites per tooth	SRP performed by a single operator	3 mo	NSPT alone	NSPT + Alpha Lipoic Acid 600 mg	–
Hong et al. (2019)	RCT, double-blind	100 (97 analysed)	35 M, 62 F	G0: 43.02 ± 14.30; G1: 37.83 ± 12.72	No uncontrolled systemic diseases; not smoking	Korean	Incipient to moderate generalized CP: PD of 4–6 mm in at least 1 site per quadrant	PD, CAL, PI, GI, REC, VAS	UNC-15; 6 sites per tooth	SRP + OHI using the same toothbrush and toothpaste	3 mo	NSPT + placebo (ND)	NSPT + vitamin C 150 mg + vitamin E 10 mg + lysozyme 30 mg + carbazochrome 2 mg	–
Rampally et al. (2019)	RCT	42	NS	(30–65)	T2DM; Taking metformin 500 mg/day	Indian	CP: At least 4 teeth with PD ≥ 5 mm, CAL ≥ 4 mm (Machtei et al. 1992)	PD, CAL, GI	Williams probe	SRP (no further details)	3 mo	NSPT + placebo (empty gelatin capsules)	NSPT + aspirin 75 mg once a day for 90 days	NSPT + Omega-3 fatty acids 500 mg twice a day for 90 days
Pelekos et al. (2019)	RCT, double-blind, parallel	59 (41 analysed)	30 M, 39F; (15 M, 26F analysed)	54.1 ± 9.0; (53.5 ± 9.6 analysed)	Systemically healthy; not smoking	Hong Kong	At least 2 non-adjacent teeth with PD ≥ 5 mm and evidence of radiographic bone loss (Armitage 1999)	PD, CAL, BOP, %PI	UNC-15; 6 sites per tooth	SRP + OHI in at least 5 sessions, performed both by hand and ultrasonic instrumentation	3, 6 mo from beginning of SRP + HM administration	NSPT + placebo (ND)	NSPT + Probiotics (*L. reuteri* 2E8 CFU) twice a day for 28 days	–
Theodoro et al. (2019)	RCT, examiner-masked, parallel	34 (28 analysed)	G0: 4F; G1: 9F	G0: 45.07 ± 6.3; G1: 47.25 ± 7.10	Systemically healthy; smokers (10 + cigarettes / day	Brazil	severe generalized CP: at least 6 teeth with PD and CAL ≥ 5 mm and at least 40% of sites with PD and CAL ≥ 4 mm and BOP + (Armitage 1999)	PD, CAL, REC, BOP%	UNC-15; 6 sites per tooth	SRP in 1 session lasting 1 h, performed by two specialists both by hand and ultrasonic instrumentation	3 mo	NSPT + placebo (ND)	NSPT + Probiotics 450 mg (*L. reuteri*, 1E8 CFU live) twice a day for 21 days	–
Soares et al. (2019)	RCT, double-blind	60	24 M, 36 F	57.0 ± 10.6	Systemically healthy	Brazilian	Stage III or IV generalized periodontitis, grade B or C (Caton et al. 2018)	PD, CAL, BOP, PI, GBI	UNC-15; 4 sites per tooth	SRP performed by a single operator both by hand and ultrasonic instrumentation	1, 2, 3 mo	NSPT + placebo (xylitol)	NSPT + xylitol + Probiotics (*L. reuteri* 10^9^ CFU / day; *L. salivarius* 10^9^ CFU / day; *L. acidophilus* 5 × 10^8^ CFU / day)	–
Vohra et al. (2020)	RCT, examiner-masked, parallel	64	64 M	G0: 51.5 ± 2.4; G1: 52.8 ± 1.6	Systemically healthy; not smoking	Saudi Arabia	CP (Tonetti et al. 2018a)	PD, CAL, %BOP, %PI	"conventional" periodontal probe; 6 sites	SRP performed by a single experienced operator, both by hand and ultrasonic instrumentation	3, 6 mo	NSPT alone	NSPT + Probiotics (*L. reuteri* 2E8 CFU) twice a day for 21 days	–
Castro Dos Santos et al. (2020)	RCT, double-blind	75 (73 analysed)	G0: 64% F; G1: 64% F; G2: 48% F	G0: 54.9 ± 9.7; G1: 55.6 ± 8.3; G2: 54.4 ± 10.2	T2DM treated with oral hypoglycemic agents and/or insulin	Brazil	Severe generalized CP: at least 6 sites with PD and CAL ≥ 5 mm and BOP + (Armitage 1999)	PD, CAL, REC, BOP, PI, inflammatory markers	Manual probe	SRP in 1 session, performed by a single experienced and trained periodontist, both by hand and ultrasonic instrumentation (with subgingival inserts)	3, 6 mo	NSPT + placebo (ND)	NSPT + Omega-3 PUFAs 900 mg + ASA 100 mg daily for 2 mo	Omega-3 PUFAs 900 mg + ASA 100 mg daily for 2 mo before periodontal treatment + NSPT
Tinto et al. (2020)	RCT, triple-blind	20	12 M, 8 F	45.6	Systemically healthy	Italian	severe stage III periodontitis: at least 4 teeth with PD ≥ 6 mm, CAL ≥ 5 mm (Tonetti et al. 2018b)	PD, BOP, PI	NS	Full-mouth one‐stage protocol (Quirynen et al. 2000): SRP performed both by hand and ultrasonic instrumentation; nearly 45 min per quadrant	6 mo	NSPT + placebo (tablets containing pregelatinized starch USP XXII, magnesium stearate, silicone dioxide, talc) for 1 mo	NSPT + Melatonin 1 mg once a day per 1 mo	–

### Risk of bias

The results of the risk of bias evaluation for studies involving healthy and systemically compromised subjects are shown in Appendix 3. Briefly, the evaluation of 17 out of 38 studies (43.6%) raised some concerns about the risk of bias, while the others (56.4%) were judged to be at low risk. The main concerns about the risk of bias evaluation were due to inadequate description of the randomization and allocation methods (16 of 38 studies, 41.0%), the number of dropouts (3 of 38 studies, 7.7%), and the absence of the placebo (8 of 38 studies, 20.5%), which might have influenced the awareness of the patient of their assigned intervention. Twenty of the included papers reported that they were supported in different forms (financial support, grant or the products) by manufacturers of the host modulators tested.

### Synthesis of the results

The summary-of-findings tables are presented in Appendix 4.

#### Omega-3

Omega-3 (PUFA n-3) were tested in four studies^31–34^, where the host modulator was administered with different prescriptions and doses (EPA 180 mg / DHA 120 mg once a day for 3 months; EPA 6.25 mg + DHA 19.19 mg twice a day for 6 months; Omega-3 fatty acids 500 mg twice a day for 90 days; PUFAs 300 mg once a day for 2 weeks). Three studies were in systemically healthy patients and one in diabetic type 2 patients taking metformin^33^.

##### Healthy patients

- Primary outcomes
  Owing to the limited number of studies, meta-analysis combined data from healthy patients^31,32^ and patients affected by type 2 diabetes^33^ and results are presented in Table 2. The differences between the test and the control groups were not statistically significant for PD and CAL. The certainty of the available evidence (GRADE) was rated as very low (Appendix 5). Another study not included in the meta-analysis in healthy subjects reported that dietary supplementation of Omega-3 had no benefit on clinical parameters.
- Secondary outcomes
  When combining data from healthy^31,32^ and diabetic type 2 patients^33^, no significant improvement in GI were observed between the test and the control groups (Table 2).
  Plaque levels, reported in the study by Deore et al.^31^, improved significantly in both groups, without any significant difference between them. Two studies on healthy patients indicated that dietary supplementation of Omega-3 reduced the levels of TNF-α^34^ and of IL-1beta^32^.
  None of the studies reported the occurrence of any complication or adverse events.

**Table 2:** Summary of the results of the meta-analysis.

	3 months				6 months				9 months				12 months			
	Mean [95% CI] (n° of studies)	*P*	*I*^*2*^	Certainty of evidence (GRADE)	Mean [95% CI] (n° of studies)	*P*	*I*^*2*^	Certainty of evidence (GRADE)	Mean [95% CI] (n° of studies)	*P*	*I*^*2*^	Certainty of evidence (GRADE)	Mean [95% CI] (n° of studies)	*P*	*I*^*2*^	Certainty of evidence (GRADE)
**Omega 3**																
PD red	0.21 [− 0.12, 0.55] (3 studies)	0.21	90%	Very Low	–	–	–	–	–	–	–	–	–	–	–	–
CAL gain	0.46 [− 0.19, 1.11] (3 studies)	0.17	97%	Very Low	–	–	–	–	–	–	–	–	–	–	–	–
GI red	0.08 [− 0.09, 0.25] (3 studies)	0.37	84%	Very Low	–	–	–	–	–	–	–	–	–	–	–	–
**Subantimicrob. Tetracycline**																
PD red	0.20 [0.00, 0.40] (5 studies)	0.05	99%	Very Low	–	–	–	–	–	–	–	–	–	–	–	–
CAL gain	**0.30 [0.19, 0.41] (4 studies)**	** < 1E**−**5**	97%	Low	–	–	–	–	–	–	–	–	–	–	–	–
**Vitamins**																
PD red	0.02 [− 0.02, 0.07]	0.33	0%	Low	–	–	–	–	–	–	–	–	–	–	–	–
CAL gain	− 0.01 [− 0.07, 0.05]	0.65	0%	Low	–	–	–	–	–	–	–	–	–	–	–	–
**Melatonin**																
PD red	–	–	–	–	**0.85 [0.46, 1.24] (3 studies)**	** < 1E**−**4**	88%	Very low	–	–	–	–	–	–	–	–
**Probiotics**																
*All species*																
PD red [*all sites*]	**0.30 [0.11, 0.48] (11 studies)**	**0.002**	93%	Low	0.20 [− 0.27, 0.68] (7 studies)	0.40	96%	Low	–	–	–	–	**0.84 [0.22, 1.46] (3 studies)**	**0.008**	95%	Low
PD red [*4–6 mm*]	**0.15 [0.02, 0.28] (3 studies)**	**0.02**	29%	Moderate	–	–	–	–	–	–	–	–	–	–	–	–
PD red [> = *7 mm*]	**0.49 [0.03, 0.96] (4 studies)**	**0.04**	66%	Low	–	–	–	–	–	–	–	–	–	–	–	–
CAL gain [*all sites*]	**0.21 [0.11, 0.31] (11 studies)**	**0.0001**	80%	Low	0.21 [− 0.15, 0.56] (7 studies)	0.25	98%	Low	–	–	–	–	**0.70 [0.36, 1.04] (3 studies)**	**0.0001**	85%	Low
CAL gain [*4–6 mm*]	**0.27 [0.05, 0.49] (3 studies)**	**0.02**	64%	Low	–	–	–	–	–	–	–	–	–	–	–	–
CAL gain [> = *7 mm*]	0.66 [− 0.08, 1.39] (4 studies)	0.08	80%	Very low	–	–	–	–	–	–	–	–	–	–	–	–
BOP% red	**6.85 [3.36, 10.34] (11 studies)**	**0.0001**	67%	Low	3.50 [− 1.46, 8.47] (7 studies)	0.17	60%	Very low	–	–	–	–	**7.41 [2.34, 12.49] (3 studies)**	**0.004**	0%	Low
PI% red	**5.00 [0.80, 9.21] (6 studies)**	**0.02**	68%	Low	1.84 [− 3.97, 7.64] (4 studies)	0.54	59%	Very low	–	–	–	–	–	–	–	–
*L. reuteri*																
PD red	**0.33 [0.08, 0.58] (6 studies)**	**0.010**	93%	Moderate	0.41 [− 0.37, 1.19]	0.31	97%	Low	–	–	–	–	–	–	–	–
CAL gain	**0.26 [0.14, 0.38] (6 studies)**	** < 1E**−**4**	85%	Moderate	0.43 [− 0.07, 0.92] (4 studies)	0.09	99%	Low	–	–	–	–	–	–	–	–
BOP% red	**5.41 [0.31, 10.56] (6 studies)**	**0.04**	69%	Low	2.24 [− 4.18, 8.65] (4 studies)	0.49	68%	Low	–	–	–	–	–	–	–	–

Bold means significanlty different (from the statistical point of view).

##### Systemically compromised patients

- Primary outcomes
  No significant differences in PPD and CAL at 3 months of healing were observed when omega-3 or a placebo were administered together with NSPT^33^.
- Secondary outcomes
  In diabetic patients, plasma level of pentraxin (PTX3) improved significantly more when omega-3 rather than low-dose aspirin or placebo were combined with NSPT^33^.

#### Omega-3 and acetylsalicylic acid

PUFA n-3 were combined with acetylsalicylic acid (ASA) in two studies, which were both on diabetic type 2 patients treated with hypoglycemic drugs and/or insulin^28,30^. The doses of ASA varied from 75 to 100 mg daily for up to 6 months.

##### Healthy patients

The literature search did not identify any RCT where NSPT was combined with Omega-3 and ASA in healthy patients.

##### Systemically compromised patients

- Primary outcomes
  Owing to the limited number of studies, the meta-analysis could not be performed.
  Remarkably, one study^30^ found that 2-month administration of omega-3 plus ASA before or after NSPT increased the number and percentage of patients that reached the endpoint for treatment (≤ 4 pockets with PD ≥ 5 mm) compared to the control patients that only received NSPT and placebo, while the other study reported a highly significative difference (*P* ≤ 0.01) for values of PD and CAL at 3 and 6 months follow-ups between the test and the control group^28^.
- Secondary outcomes
  While in one study inflammation scores (BOP% and GI) showed a similar improvement in the test and control groups, without significant inter-group differences^30^, the other study found a statistically significant difference in GI between groups at 3 and 6 months^28^.
  Dos Santos et al.^30^ showed that plaque level decreased significantly more when NSPT was combined with omega-3 plus low-dose aspirin for 2 months after NSPT. The authors also indicated that cytokine levels inversely correlated with periodontal parameters when adjunctive omega-3 PUFA and ASA therapy was administered, as opposed to the positive correlation detected in the placebo group.
  Elwakeel and Hazaa reported nausea, abdominal upsets and irritating fish-scented halitosis in 13 out of 20 subjects in the intervention group^28^.

#### NSAIDs

Only 2 studies were identified, one in healthy subjects and one in type 2 diabetic patients, so no meta-analysis could be performed.

##### Healthy patients

- Primary outcomes
  One study tested the effect of cyclooxygenase-2 inhibitor (Celecoxib 200 mg daily for 6 months) as an adjunct to NSPT in systemically healthy patients and it showed significant improvements in both PD and CAL in the test compared to the control group^35^. This beneficial effect appeared to be more evident in pockets with baseline PD ≥ 7 mm, having PD reduced of 3.27 ± 1.56 mm in the test group and of 1.89 ± 1.60 mm in the control group after 3 months^35^.
- Secondary outcomes
  Plaque level and BOP decreased similarly in patients that received Celocoxib associated with NSPT or not.
  The authors indicated no concerns about drug safety^35^, and complications / adverse effects were not reported.

##### Systemically compromised patients

- Primary outcomes
  A recent study on adjunctive administration of ASA to NSPT in subjects with type 2 diabetes failed to demonstrate a beneficial effect of this HM^33^.
- Secondary outcomes
  GI decreased similarly in patients that received ASA or not. No complications/adverse events were reported.

#### Melatonin

Melatonin was studied as an adjunctive HM to NSPT in three studies^36–38^ in systemically healthy patients. Different dosages (1 mg, 2 mg, 10 mg), and different administration periods (up to 2 months) were adopted.

##### Healthy patients

- Primary outcomes
  The 6-month PD reduction was significantly different between the two groups (0.85 mm, 95%CI 0.46 mm to 1.24 mm), with high heterogeneity among the studies (Table 2). The certainty of evidence (GRADE) was very low (Appendix 5).
- Secondary outcomes
  In two studies, gingival bleeding level (BOP%), as well as plaque levels decreased similarly in both test and control groups^36,38^.
  In two studies few subjects reported minor adverse reactions, such as headache, dizziness, nausea, constipation, diarrhea, and abdominal cramp (2 cases)^36,37^.

##### Systemically compromised patients

The literature search did not identify any RCT where NSPT was combined with melatonin in systemically compromised subjects.

#### Biphosphonates

Two studies reported data about the systemic adjunctive administration of bisphosphonates (alendronate and neridronate) to NSPT in healthy patients^20,39^, therefore no meta-analysis could be performed.

##### Healthy patients

- Primary outcomes
  One study assessed the adjunctive administration of 10 mg alendronate (for 6 months) in postmenopausal women, reporting significantly higher improvements in clinical parameters in the test group (PD reduction of 0.8 ± 0.3 mm and CAL gain of 0.99 ± 0.8 mm) than in the control group (PD reduction of 0.4 ± 0.4 mm and CAL gain of 0.5 ± 0.8 mm) at the 6-month follow-up^20^. Another study tested the adjunctive effect of 12.5 mg neridronate (once a week for 12 weeks) to NSPT^39^, but no significant improvement was observed in the short term (6 months after the beginning of the treatment).
- Secondary outcomes
  In one study the authors reported a significant improvement in gingival bleeding (BOP%) in the test group^20^, whilst another study did not find any difference in full-mouth bleeding values changes between groups^39^.
  In both studies, the improvement in plaque level was not affected by the administration of bisphosphonates.
  In one study the authors reported that eight subjects in the test group experienced unspecified adverse events^39^.

##### Systemically compromised patients

The literature search did not identify any relevant RCT where NSPT was combined with bisphosphonates in systemically compromised subjects.

#### Vitamins

Either vitamin complexes or single products were studied as adjunctive host modulators in four studies in healthy patients^40–43^.

##### Healthy patients

- Primary outcomes
  No statistically significant differences were found for PD and CAL changes at 3 months of follow-up (Table 2). The quality of evidence (GRADE) for these comparisons was low (Appendix 5).
- Secondary outcomes
  The bleeding levels changes after treatment were comparable between test and control groups^40,41,43^. Likewise, no inter-group differences were found in terms of plaque levels changes^40,42^.
  Another study found that combining NSPT with Vitamin E supplementation improved superoxide dismutase activity in serum^43^.
  Only one study in this group reported explicitly no complications ^42^, while the others did not provide any information about it. The study by Hong reported a significant improvement in the test group of patients’ self-reported gingival comfort, as evaluated by one questionnaire^42^.

##### Systemically compromised patients

The literature search did not identify any relevant any relevant RCT where NSPT was combined with vitamins in systemically compromised subjects.

#### Probiotics

A total of 11 studies tested the adjunctive effect of probiotics to NSPT in healthy patients. Six studies used *Lactobacillus reuteri* alone^24,26,44–47^, one combined it with *Lactobacillus salivarius* and *Lactobacillus acidophilus*^48^, two studies tested *Lactobacillus rhamnosus*^49,50^, one study employed *Streptococcus oralis*, *Streptococcus uberis*, and *Streptococcus rattus*^51^ and one administered *Bifidobacterium lactis*^52^.

##### Healthy patients

- Primary outcomes
  Meta-analysis included all the 11 studies in this group, and the results are presented in Table 2. At 3 months a significant benefit in terms of PD reduction and CAL gain was observed when using probiotics (0.30 mm, 95%CI 0.11 mm to 0.48 mm and 0.21 mm. 95% CI 0.11 mm to 0.31 mm, respectively), while at 6 months no significant benefit was observed. The 3 studies that reported data at 12 months indicated an increased reduction of PD (0.84 mm; 95%CI 0.22 mm to 1.46 mm) and CAL gain (0.70 mm; 95% CI 0.36 mm to 1.04 mm) when probiotics were combined with NSPT. At all time points, the quality of evidence (GRADE) was rated as low (Appendix 5).
  When considering studies that stratified the results based on PD, the use of probiotics seemed to be more beneficial in deep sites (PD ≥ 7 mm), although the quality of evidence (GRADE) was rated as very low / low (Appendix 5).
  A sub-analysis of studies testing *L. reuteri* alone was performed (Table 2). A significant improvement in terms of PD and CAL (0.33 mm; 95% CI 0.08 mm to 0.58 mm and 0.26 mm; 95% CI 0.14 mm to 0.38 mm) was observed at 3 months and the quality of evidence (GRADE) for such product was considered as moderate. At 6 months no significant differences could be obtained when using or not this specific probiotic combined with NSPT.
- Secondary outcomes
  The adjunctive use of probiotics improved BOP and PI at 3 months (6.85%; 95% CI 3.36% to 10.34% and 5%; 95% CI 0.80% to 9.21%, respectively) and BOP at 12 months (7.41%; 95% CI 2.34% to 12.49%). However, the quality of evidence (GRADE) was rated as low for both parameters (Appendix 5).
  When looking at studies testing *L. reuteri* alone associated with NSPT, a significant improvement in BOP was reported (5.41%; 95% CI 0.31% to 10.56%), but the quality of evidence (GRADE) was rated as low (Appendix 5).
  Only one study in this group reported one minor complication in the control group (one patient referred unspecified “discomfort”)^45^.

##### Systemically compromised patients

The literature search did not identify any relevant any relevant RCT where NSPT was combined with probiotics in systemically compromised subjects.

#### Sub-antimicrobial dose of tetracycline (SDD)

Ten studies tested the systemic administration of sub-antimicrobial doses of tetracycline (SDD) as adjunct to NSPT^21–23,25,27,52–57^. In one study the authors tested incyclinide^53^ while in all the other studies the authors administered doxycycline, with various regimens. All studies involved systemically healthy patients, apart from one study that recruited type 2 diabetic patients^27^.

##### Healthy patients

- Primary outcomes
  A benefit in PD reduction and CAL gain was observed at 3 months when adding SSD to NSPT (0.20 mm; 95% CI 0.00 mm to 0.40 mm and 0.30 mm; 95% CI 0.19 mm to 0.41 mm, respectively) in systemically healthy patients (Table 2), with the quality of evidence (GRADE) rated as very low / low (Appendix 5). The study published by Needleman et al.^23^, not included in the meta-analysis, tested doxycycline on a cohort of smokers, without finding any clinical or biochemical markers advantage in the test group.
  In three studies, the authors provided data stratified on the basis of initial PD. Mohammad et al.^21^ reported a significantly higher PD reduction and CAL gain when administering SDD both in moderate pockets (4–6 mm) and in deeper ones, for all timepoints (3, 6, and 9 months). Likewise, in two separate studies on large samples of subjects (209 and 227 respectively), it was found that PD reduction and CAL gain were significantly improved in the test compared to the control group, with better results in deeper pockets than in moderate ones^22,57^.
- Secondary outcomes
  Few studies reported a significantly higher decrease in gingival inflammation when SDD was administered^21,22,25,56^. Plaque levels tended to decrease in a similar way (without any significant difference) between test and control groups^25,53,54^.
  Three studies reported the occurrence of adverse effects. In particular, one study reported adverse events in five subjects belonging to the control group, probably not related to the treatment^23^ and another study reported that seven subjects in the control group quitted the study due to the occurrence of adverse events^57^. Preshaw et al.^22^ indicated a total of 217 and 229 adverse events in the test and control groups, respectively. In the SDD-treated group the most frequently reported adverse events were headache, influenza and naso-pharyngitis, while in the placebo group the most frequently reported adverse events were sensitivity of teeth, headache and naso-pharyngitis. No severe adverse events were considered related to the treatment.

##### Systemically compromised patients

- Primary outcomes

Gilowsky et al.^27^ showed a significant difference in PD reduction between diabetic type 2 patients receiving SDD and patients receiving the placebo after 3 months from NSPT when considering sites with initial moderate disease (PD ≥ 4 mm).

- Secondary outcomes

While BOP improved after NSPT, no significant differences were detectable between diabetic type 2 patients receiving SDD or not^27^. GCF matrix metalloproteinase-8 levels were significantly reduced only in SRP + SDD group 3 months after therapy.

#### Others

One study evaluated the adjunctive administration of Alpha Lipoic Acid (ALA) in 40 (20 per group) subjects with periodontitis and type 2 diabetes mellitus^29^. The results demonstrated a significant effect of ALA in improving both PD and CAL, as well as GI after 3 months of treatment. Moreover, Surapanemi et al.^29^ reported that the administration of ALA after NSPT could reduce the levels of serum resistin and HbA1c in diabetic patients.

## Discussion

The present systematic review evaluated the effect of the adjunctive systemic administration of HMs on the outcomes of NSPT and it indicated, as evaluated by GRADE approach, an overall low/very low quality of evidence for SDD and melatonin in improving PD and/or CAL gain when administered in systemically healthy patients. Conflicting evidence is available for probiotics administered in systemically healthy patients, with low evidence of a benefit at 3 and 12 months but no significant benefit at 6 months post NSPT. The dosage, posology and long-term effect of HMs still need to be clarified. It should be noted that only 5 studies dealt with systemically compromised patients and they all included type 2 diabetic patients, so no speculation can be done on the potential benefit of HMs in patients with underlying medical conditions associated with an altered/exaggerated inflammatory response other than diabetes.

In particular, meta-analysis indicated that there is low/very low evidence that the adjunctive use of SDD would lead to a significant improvement both in terms of CAL (0.30 mm) and PD (0.20 mm) in the short term (3 months), although this benefit cannot be considered as clinically relevant. No meta-analysis could be performed for longer healing times, nevertheless few studies suggested a benefit up to 9 months post NSPT, particularly in case of deep pockets (≥ 7 mm)^25,57,58^. Only one study assessed SDD in diabetic type 2 patients and it suggested a significant difference in PD reduction between patients receiving SDD and patients receiving the placebo after 3 months from NSPT n sites with PD ≥ 4 mm^27^.

While the use of omega-3 alone did not provide a significant benefit when added to NSPT, the combination with low-dose aspirin significantly improved both PPD reduction and CAL gain both at 3- and 6-months post NSPT as reported in two studies^28,30^. It should be noted that the studies testing this combination involved patients affected by diabetes type 2, thus suggesting that this particular subgroup of patients might specifically benefit from the addition of modulators of the inflammatory response, although the current quality of evidence is very low, and we could not perform a meta-analysis. Remarkably, one additional study, not included in the review for methodological concerns regarding the allocation method, reported a substantially positive effect of the adjunctive assumptions of omega-3 and ASA in a cohort of systemically healthy subjects^59^.

When analyzing the use of different probiotic preparations, there was low-grade evidence that they would improve PD reduction and CAL gain at 3 (0.30 mm and 0.21 mm, respectively) and 12 months (0.84 mm and 0.70 mm, respectively) post NSPT in systemically healthy patients, particularly in deep pockets (≥ 7 mm), while no benefits were observed at 6 months, thus confirming previous findings^8^. A sub-analysis of 6 studies testing *L. reuteri* alone was performed, and there was moderate evidence of a significant effect for PD reduction and CAL gain after three months, but the evidence was judged as low for all other outcomes and time points.

Moreover, 3 studies suggested a benefit in PD reduction when melatonin was combined with NSPT in systemically healthy patients, however the clinical benefit was limited (0.85 mm) and the overall quality of the available evidence was judged as very low. No significant benefit was associated with the use of vitamins and insufficient data were available for other HMs. In line with what recently recommended by the EFP S3 level clinical practice guideline and considering the well-documented risk of severe adverse events associated with the systemic use of bisphosphonates and NSAIDs, it is not recommended to use these systemic HMs to enhance the outcomes of NSPT.

Our outcomes corroborated the results of a recent systematic review^8^, but also added important additional and complementary information in terms of the effect of HMs on early healing (3 months), grade of the evidence and role of HMs in the presence of systemic diseases (e.g., diabetes).

The early healing (3 months post NSPT) response after delivery of a HM was considered important to investigate since it may be less affected by patient’s compliance to oral hygiene instructions and, therefore, might provide relevant information on the “true” potential of the modulator.

It is worth highlighting that in this systematic review we performed meta-analysis when ≥ 3 studies investigating the same HM were available. While a meta-analysis is simply the statistical combination of results and there is no fixed number of studies or combined number of individuals that can be used as a threshold to decide whether data are warrant statistical combination^60–62^, we recognize that some level of caution needs to be applied when drawing conclusions based only on few studies, in particular when the studies are heterogeneous. Remarkably, besides providing meta-analyses, this systematic review was one of the first in the field of periodontology to include also an assessment of the strength of the evidence for each comparison and for each outcome considered according to the GRADE system. As a matter of fact, when elaborating evidence-based treatment guidelines, it is of outmost importance to evaluate not only the statistical significance of a summary estimate of treatment effect and the effect size, but also the quality and confidence in that estimate. The purpose of GRADE is to offer a transparent, user-friendly and pragmatic tool that clearly separates between quality of evidence and strength of recommendations and is therefore a valuable system to support clinicians in decision-making about healthcare^63^ and it has already been adopted by several international associations involved in the development of treatment guidelines, such as the World Health Organization, the American College of Physicians and the Cochrane Collaboration^19^. An insufficient attention to quality of evidence exposes clinicians and researchers to the risk of developing inappropriate/misleading guidelines and recommendations that act to the detriment rather than to the benefit of their patients.

Overall, the GRADE assessment revealed that the quality of evidence in the investigated field is low or very low and such evaluation was mainly due to the significant heterogeneity among studies, differences in treatment protocols and risk of bias. In particular, the different dosages administered in the SDD group and the different probiotic formulations tested have reduced the scientific evidence for these HMs.

While host modulators can possibly enhance the outcomes of NSPT in all patients, it is reasonable to hypothesize that they might become particularly useful in patients that have an exaggerated/ineffective inflammatory-immune response because of an underlying medical condition. Diabetes type 2 is an example of multifactorial disease in which inflammation plays a crucial role in promoting insulin resistance and the development of long-term complications and has a well-recognized link with periodontitis, so that periodontitis is even considered its 6^th^ most frequent complication^64^. Few studies suggested that controlling the inflammatory response with a HM that can actively promote inflammation resolution (like alpha lipoic acid or the combination of omega-3 and aspirin) or downregulates the activity of matrix metalloproteinases (SDD)^27^, can enhance the clinical outcomes of NSPT^28–30,33^. However, the current evidence is too limited to draw any robust conclusion on the potential of HMs as adjunct to NSTP in systemically compromised conditions and no data were retrieved from the identified papers on the benefit of HMs on other potentially relevant diseases like rheumatoid arthritis or osteoporosis, which would warrant further investigations.

When translating the outcomes of this review and meta-analysis to clinical practice, a certain level of caution needs to be adopted. While overall no serious adverse events were reported by any of the included studies, the recent S3 treatment guidelines for periodontitis stage I–III raised some concerns on the clinical use of SDD for periodontal patients due to current health policies on antibiotic stewardship and related public health concerns surrounding the global problem of antibiotic resistance^4^. Hence, in consideration of this important potential issue and the limited clinical efficacy of this HM, it is currently suggested not to use SDD as an adjunct to NSPT.

Moreover, the primary outcomes selected in this systematic review were PPD reduction and CAL gain, which are the most commonly reported surrogate outcomes in studies on periodontal treatment, despite they present with several limitations^65^. Considering that the main goal of periodontal therapy is to achieve shallow pockets and absence of bleeding, the percentage of pocket closure could have been possibly a more valuable outcome to assess the performance of HMs^66^, but only a minority of the selected studies evaluated it. It is also suggested that with the aim of developing guidelines for periodontal therapy the proportion of threshold changes such as ≥ 2 mm or ≥ 3 mm in clinical attachment levels are preferable rather than mean changes^66^, but again only a minority of the included studies provided data in this respect. It should be noted that, since the rational of using of HMs has to do mainly with the modulation of the exaggerated immune-inflammatory response towards the microbial challenge, inflammatory indices (such as bleeding scores and gingival indices) should be taken into consideration when assessing the treatment response. Overall, all HMs tended to reduce the levels of inflammation compared to the placebo or no treatment, but due to the heterogeneity in the indices measured, meta-analysis could only be performed for probiotics and it indicated a significant reduction in BOP at 3 and 12 months (6.85% ad 7.41%, respectively) when probiotics were combined with NSPT.

One of the potential limitations related to the studies included in this systematic review is that they all aggregated patient-level data providing a summary statistical approach (i.e., mean) for PD and CAL changes. While this allows to assess an overall effect of the different HMs on the periodontal condition of the patient, it should be recognized that periodontitis is most often a site-specific disease, and by aggregating site-level evaluations there is the risk of losing important information^67^ and diluting the real effect that the HM might have had. In this respect, it is interesting to mention that Pelekos et al.^68^ have recently performed a sub-analysis of site-level data sourced from a previously published study^45^ where they showed that, while a 4-week administration of *L. reuteri* did not provide benefits on aggregate patient-level outcomes, a significant modest benefit in terms of CAL gain could be expected when focusing only on molar sites with PD ≥ 5 mm. Moreover, in these sites the relative risk of pocket closure was higher in the probiotic group than in the placebo group (1.7 at 90 days and 1.6 at 180 days). Likewise, studies that stratified the treatment response to SDD according to the initial PD depth showed an enhanced PD reduction and CAL gain when focusing only on pockets that had a baseline PD ≥ 7 mm^21,22,25,57,58,69,70^. It is therefore suggested that future studies testing systemic MDs should perform multilevel analyses to assess not only the patient-level but also the site-level response to them. In particular, it would be interesting to explore if HMs have a positive impact on the treatment response of particularly challenging sites, like deep pockets associated with intrabony defects or furcation involvement. Remarkably, Donos et al.^8^ have shown that locally delivered HMs (namely statins, bisphosphonates and metformin) can significantly improve the response to NSPT of deep vertical intrabony defects.

Another limitation that should be mentioned when analyzing the results of the present systematic review is that the protocol for NSPT adopted in the different studies was not consistent. While some studies did not provide details on how the instrumentation was performed, other studies reported a different number of sessions, a different length of visits and possibly a different level of experience of the operators. Hence, it is not possible to assess if the different NSPT protocols impacted on the clinical outcomes. Moreover, the study populations differed in terms of systemic health status, smoking status, age and gender distribution and we cannot make any conclusions on how these factors might have influenced the outcomes investigated.

Finally, it is worth to highlight that the great majority of the studies were conducted in a controlled academic/hospital environment by researchers that possibly had a level of training, skills and attention to NSPT that might not reflect the average level of general dentists, so the studies informed more on the efficacy rather than effectiveness of HMs. It would be important in the future to test the most promising HMs at a primary care level to assess their effectiveness rather than efficacy.

## Conclusions

There is low/very low evidence based on the results of RCTs that the adjunctive use of SDD and melatonin to NSPT would lead to a statistically significant improvement in clinical periodontal parameters, while conflicting evidence is available on the efficacy of probiotics. Owing to the heterogeneity of the available studies and the limited average clinical benefit indicated by the meta-analyses, currently there is no robust evidence to suggest the implementation of any of the aforementioned HMs in clinical practice. The potential benefit of HMs in systemically compromised patients affected by periodontitis needs to be further investigated, as currently there is only some limited evidence on type 2 diabetes and none of the identified RCTs dealt with other systemic diseases. In particular, the combination of omega-3 and low dose aspirin as an adjunct to NSPT gave promising outcomes in type 2 diabetic patients, which need to be conformed by further RCTs.

Future studies controlling for confounding factors and using composite outcomes to define the endpoint of therapy are warranted. Moreover, it is recommended that not only the patient level but also as the site-specific effect of systemic HMs is clarified.

## Supplementary Information

Supplementary Information 1.

## Data Availability

The authors confirm that the data supporting the findings of this study are available within the article or its supplementary materials.
