# Supplementary material for: Systematic review and meta-analysis on the adjunctive use of host immune modulators in non-surgical periodontal treatment in healthy and systemically compromised patients
Source: Sci Rep. 2021 Jun 9;11:12125. doi: 10.1038/s41598-021-91506-7 (PMC8190303; doi:10.1038/s41598-021-91506-7)
Supplement: Supplementary file 1 — Supplementary Information 1. [file 41598_2021_91506_MOESM1_ESM.docx]

**Appendices**

***Appendix 1***

1. Search strategy for OVID

| Medline via OVID | | |
| --- | --- | --- |
|  | MeSH terms | Free text |
| Population | Exp Periodontal Diseases/ | Periodont$ or parodontos$ or pyorrhea or periodontal pocket* |
| Intervention/ Exposure | Exp anti-inflammatory agents, non-steroidal/ OR  Exp Fatty Acids, unsatured OR  Exp Diphosphonates/ OR  Hydroxymethylglutaryl-CoA Reductase Inhibitors/ Or Exp metformin OR exp vitamins or micronutrients OR exp trace elements OR prebiotics/ OR probiotics/ OR exp fish oils OR exp immunomodulation OR aloe/ OR doxycycline/ OR plants, medicinal/ | NSAIDs or ((Anti adj2 inflammatory) and (agent or analgesic)) or (non adj2 steroidal) or fenoprofen or ibuprofen or indoprofen or ketoprofen or suprofen or diclofenac or flurbiprofen or peroxicam or coxib or (n-3 adj2 fatty acid) or PUFA or (n-3 adj2 oil) or polyunsatured fatty acid or lipoxin or maresin or protectin or resolving or bisphosphonate or bisphosphonate or diphosphonate or alendron$ or clodron$ or bonefos or CI2MDP or dichloromethane& or etidron$ or Fosamax or MK217 or EHDP or HEDP or phosphonic acid or statin or anticholester$ or (cholesterol adj2 inhibitor) or hypocholester$ or (Hydroxymethylglutaryl adj2 CoA) or (Hydroxymethylglutaryl adj2 coenzyme A) or (host adj2 modulator) OR immunomodulator* OR doxycycline OR (aloe adj2 vera) OR curcumin OR (turmeric adj2 yellow) OR resveratrol OR (plant AND preparation$) OR (herbal AND preparation$) OR tea OR (plant AND extract$) OR (plant AND oil$) OR melatonin OR indoleamine OR metformin OR vitamin |

This subject search was linked to the Cochrane Highly Sensitive Search Strategy (CHSSS) for identifying randomised trials in MEDLINE: sensitivity‐ maximising version (2008 revision) as referenced in Chapter 6.4.11.1 and detailed in box 6.4.c of The Cochrane Handbook for Systematic Reviews of Interventions, Version 5.1.0 (updated March 2011).

1. randomized controlled trial.pt.

2. controlled clinical trial.pt.

3. randomized.ab.

4. placebo.ab.

5. drug therapy.fs.

6. randomly.ab.

7. trial.ab.

8. groups.ab.

9. or/1‐8

10. exp animals/ not humans.sh.

11. 9 not 10

2. Search strategy for EMBASE

| EMBASE | | |
| --- | --- | --- |
|  | Emtree terms | Free text |
| Population | Periodontal Disease OR Exp periodontitis OR alveolar bone loss | Periodont$ or parodontos$ or pyorrhea or periodontal pocket* |
| Intervention/ Exposure | Exp nonsteroid antiinflammatory agent OR  Exp Unsatured fatty acid OR  Exp phosphorus acid derivative OR  Exp hydroxymethylglutaryl coenzyme A reductase inhibitor OR exp probiotic agent OR exp prebiotic agent OR exp trace element OR exp fish oil OR exp immunomodulation OR exp immunomodulating agent OR exp resveratrol OR exp aloe OR exp plant medicinal product OR exp curcumin OR exp melatonin OR exp metformin | NSAIDs or ((Anti and inflammatory) and (agent or analgesic)) or (non and steroidal) or fenoprofen or ibuprofen or indoprofen or ketoprofen or suprofen or diclofenac or flurbiprofen or peroxicam or coxib or (n-3 and fatty acid) or PUFA or (n-3 and oil) or “polyunsatured fatty acid” or lipoxin or maresin or protectin or resolving or bisphosphonate* or bisphosphonate* or diphosphonate* or alendron$ or clodron$ or bonefos or CI2MDP or dichloromethane$ or etidron$ or Fosamax or MK217 or EHDP or HEDP or phosphonic acid or statin or anticholester$ or (cholesterol and inhibitor) or hypocholester$ or (Hydroxymethylglutaryl and CoA) or (Hydroxymethylglutaryl and coenzyme A) or (host and modulator) or immunomodulator* or doxycycline OR aloe vera OR curcumin OR (turmeric and yellow) OR resveratrol OR (plant AND preparation$) OR (herbal AND preparation$) OR tea OR (plant AND extract$) OR (plant AND oil$) OR melatonin OR indoleamine OR metformin OR vitamin |

This subject search was linked to the RCT strategy based on SIGN filter3, amended to embase.com format:

'clinical trial'/de OR 'randomized controlled trial'/de OR 'randomization'/de OR 'single blind procedure'/de OR 'double blind procedure'/de OR 'crossover procedure'/de OR 'placebo'/de OR 'prospective study'/de OR 'randomi?ed controlled' NEXT/1 trial* OR rct OR 'randomly allocated' OR 'allocated randomly' OR 'random allocation' OR allocated NEAR/2 random OR single NEXT/1 blind* OR double NEXT/1 blind* OR (treble OR triple) NEAR/1 blind* OR placebo*

***Appendix 2***

Reasons for study exclusion.

| **Authors** | **Year of publication** | **Reason for exclusion** |
| --- | --- | --- |
| Alptekin et al. | 2000 | Not RCT |
| Alsheri | 2015 | Local delivery of host modulator |
| Alsheri, Javed | 2015 | Outcome (PD or CAL) not reported |
| Amoian et al. | 2017 | Not in English |
| Anuradha | 2015 | Local delivery of host modulator |
| Aras et al. | 2007 | Follow-up < 3 months |
| Azoubel et al. | 2008 | Follow-up < 3 months |
| Babaei et al. | 2018 | Follow-up < 3 months |
| Bazyar et al. | 2019 | Follow-up < 3 months |
| Bhavsar | 2016 | Absence of negative control group |
| Booth et al. | 1996 | Local delivery of host modulator;  Administration of antibiotics (metronidazole);  Absence of full-mouth clinical data |
| Bretz | 2012 | Not RCT |
| Bunduneli | 2010 | Follow-up < 3 months;  Absence of full-mouth clinical data |
| Caton et al. | 2000 | Absence of full-mouth clinical data |
| Caton et al. | 2001 | Absence of full-mouth clinical data |
| Choi et al. | 2004 | Absence of full-mouth clinical data |
| Ciancio et al. | 1998 | Not RCT |
| Crout | 1996 | Absence of full-mouth clinical data |
| Deshpande | 2013 | Local delivery of host modulator |
| Dodington et al | 2015 | Not RCT |
| Dutra | 2017 | Split-mouth design |
| El-Sharkawi et al. | 2010 | Not RCT |
| El Shinnawi | 2003 | Periodontotitis not defined |
| Elgendy | 2018 | Absence of full-mouth clinical data |
| Emingil et al. | 2004 | Study reporting potentially duplicated data |
| Emingil et al. | 2008 | Study reporting potentially duplicated data |
| Emingil et al. | 2011 | Study reporting potentially duplicated data |
| Emingil et al. | 2019 | Study reporting potentially duplicated data |
| Engebretson | 2011 | Outcome (PD or CAL) not reported |
| Fajardo et al. | 2010 | Absence of negative control group |
| Flemmig | 1996 | Split-mouth design |
| Funosas | 2009 | Local delivery of host modulator |
| Garg | 2017 | Local delivery of host modulator |
| Goel | 2016 | Local delivery of host modulator |
| Golub | 2001 | Absence of full-mouth clinical data |
| Graziani et al. | 2018 | Not about host modulators / Concentrations not defined |
| Gurkan et al. | 2005 | Study reporting potentially duplicated data |
| Gurkan et al. | 2008 | Study reporting potentially duplicated data |
| Haffaje et al. | 2008 | Outcome (PD or CAL) not reported |
| Haffajee et al. | 1996 | Surgical treatment included;  Administration of antibiotics (tetracycline) |
| Haffajee et al. | 1995 | Surgical treatment included;  Administration of antibiotics (tetracycline) |
| Haffajee et al. | 2007 | Administration of antibiotics |
| Heasman et al. | 1993 | Local delivery of host modulator |
| Hrishi et al. | 2015 | Local delivery of host modulator |
| Jeffcoat et al. | 1995 | Local delivery of host modulator |
| Jeffcoat et al. | 1988 | NSPT not administered |
| Kassem et al. | 2014 | Local delivery of host modulator |
| Kharaeva et al. | 2016 | Local delivery of host modulator |
| Konuganti & Kumar | 2016 | Local delivery of host modulator |
| Koppikar, Agrawal | 2013 | Outcome (PD or CAL) not reported |
| Kurtis et al. (A) | 2017 | Not RCT |
| Kurtis et al. (B) | 2017 | Not RCT |
| Lane et al. | 2005 | Absence of negative control group |
| Machtei et al. | 2011 | Local delivery of host modulator |
| Marawar et al. | 2014 | Outcome (PD or CAL) not reported |
| Martinez et al. | 2014 | Outcome (PD or CAL) not reported |
| Mohiuddin et al. | 2011 | Local delivery of host modulator |
| Murugesan et al. | 2018 | Absence of negative control group |
| Nagasri et al. | 2015 | Local delivery of host modulator;  Split-mouth design |
| Naqvi et al. | 2014 | Absence of negative control group |
| Naqvi et al. | 2017 | Absence of negative control group |
| Nasra et al. | 2017 | Local delivery of host modulator |
| Ng & Bissada | 1998 | Split-mouth design |
| Novak et al. | 2002 | Not RCT |
| Oduncuoglu et al. | 2019 | Absence of full-mouth clinical data |
| Ozden | 2017 | Not RCT |
| Ozgoren et al. | 2014 | Absence of full-mouth clinical data;  Follow-up < 3 months |
| Payne et al | 2013 | Not RCT |
| Payne et al. | 2011 | Outcome (PD or CAL) not reported |
| Pinho et al. | 2008 | Follow-up < 3 months |
| Piyushshah | 2013 | Absence of negative control group |
| Pradeep et al. | 2016 | Local delivery of host modulator |
| Pradeep et al. | 2015 | Local delivery of host modulator |
| Pradeep et al. | 2013 (a) | Local delivery of host modulator |
| Pradeep et al. | 2013 (b) | Local delivery of host modulator |
| Pradeep et al. | 2013 (c) | Local delivery of host modulator |
| Pradeep et al. | 2012 (a) | Local delivery of host modulator |
| Pradeep et al. | 2012(b) | Local delivery of host modulator |
| Pradeep et al. | 2010 | Local delivery of host modulator |
| Priyanka et al. | 2017 | Local delivery of host modulator |
| Rao et al. | 2013 | Local delivery of host modulator |
| Rasperini et al. | 2019 | Administration of antibiotics (metronidazole, amoxicillin) |
| Rath et al. | 2012 | Local delivery of host modulator |
| Reddy et al. | 1993 | Absence of full-mouth clinical data |
| Reddy et al. | 2005 | Surgical treatment included;  Local delivery of host modulator |
| Reinhardt et al. | 2007 | Absence of full-mouth clinical data |
| Renvert et al. | 2009 | NSPT not administered;  Follow-up < 3 months |
| Rocha et al. | 2001 | Absence of negative control group |
| Rosenberg et al. | 2015 | Local delivery of host modulator |
| Rosenstein et al. | 2003 | NSPT not administered |
| Sato et al. | 1988 | Local delivery of host modulator |
| Sravya et al. | 2019 | Concentrations of host modulator not defined |
| Shah et al. | 2017 | Absence of negative control group |
| Sharma et al. | 2017 | Local delivery of host modulator |
| Sharma et al. | 2012 (a) | Local delivery of host modulator |
| Sharma et al. | 2012 (b) | Local delivery of host modulator |
| Shiloah et al. | 2014 | Outcome (PD or CAL) not reported |
| Srinivas et al. | 2011 | Local delivery of host modulator |
| Subramanian et al. | 2013 | Outcome (PD or CAL) not reported; use of 3D imaging |
| Surve et al. | 2015 | Outcome (PD or CAL) not reported |
| Szkaradkiewicz et al. | 2014 | Not RCT |
| Taiyeb Ali et al. | 1993 | Split-mouth design |
| Taleghani et al. | 2018 | Not about host modulators (green tea)/ Concentrations not defined |
| Toker et al. | 2006 | Follow-up < 3 months |
| Watanabe et al. | 1984 | Invalid outcome |
| Watanabe et al. | 1988 | Invalid outcome |
| Williams et al. | 1989 | Outcome (PD or CAL) not reported |
| Zare Javid et al.  (Impact of cranberry juice enriched with W-3 and their individual consumption adjunct with nonsurgical periodontal treatment on lipid profile in diabetic patients with periodontal disease) | 2014 | Outcome (PD or CAL) not reported |
| Zare Javid et al.  (doi:10.1080/07315724.2017.1357509) | 2017 | Absence of full-mouth clinical data |
| Zare Javid et al.  (doi:10.1016/j.dsx.2019.07.042) | 2019 (a) | Follow-up < 3 months |
| Zare Javid et al.  (doi:10.2147/DMSO.S214333) | 2019 (b) | Follow-up < 3 months |
| Zhu et al. | 2017 | Absence of negative control group;  periodontitis not defined |

***Appendix 3***

Risk of bias summary, reporting the judgements about each risk of bias item for the included studies.

| **Study** | **Year** | **Overall** | **Reason** | **Bias arising from the randomization process** | **Bias due to deviations from intended interventions** | **Bias due to missing outcome data** | **Bias in measurement of the outcome** | **Bias in selection of the reported results** | **Funding bias** |
| --- | --- | --- | --- | --- | --- | --- | --- | --- | --- |
| Lee et al. | 2004 | SOME CONCERNS | Allocation concealment not clear | SOME CONCERNS | LOW | LOW | LOW | LOW | The manufacturer supported the study |
| Preshaw et al. | 2004 | SOME CONCERNS | Allocation concealment not clear; Number of droputs | SOME CONCERNS | LOW | LOW | LOW | LOW | The manufacturer of the drug supported the study |
| Rocha et al. | 2004 | SOME CONCERNS | No information about allocation / randomization | SOME CONCERNS | LOW | LOW | LOW | LOW | The manufacturer provided the study products |
| Mohammad et al. | 2005 | SOME CONCERNS | Allocation concealment not clear | SOME CONCERNS | LOW | LOW | LOW | LOW | The manufacturer of the probiotic supported the study |
| Emingil et al. | 2006 | SOME CONCERNS | Allocation concealment not clear | SOME CONCERNS | LOW | LOW | LOW | LOW | None |
| Gorska & Nedzi-Gora | 2006 | SOME CONCERNS | No information about allocation / randomization; Absence of placebo | SOME CONCERNS | SOME CONCERNS | LOW | LOW | LOW | Not declared |
| Needleman et al. | 2007 | LOW | - | LOW | LOW | LOW | LOW | LOW | The manufacturer of the drug supported the study |
| Alec Yen et al. | 2008 | LOW | Number of dropouts for some follow-up | LOW | LOW | LOW | LOW | LOW | The manufacturer of the drug supported the study |
| Preshaw et al. | 2008 | LOW | - | LOW | LOW | LOW | LOW | LOW | The manufacturer of the drug provided a gift grant; M. Bradshaw had been a paid consultant, C. Walker was currently a consultant for the manufacturer of the drug |
| Graziani et al. | 2009 | SOME CONCERNS | Absence of placebo | LOW | SOME CONCERNS | LOW | LOW | LOW | The manufacturer of the probiotic supported the study |
| Sulaiman & Shehadeh | 2010 | SOME CONCERNS | Absence of placebo | LOW | SOME CONCERNS | LOW | LOW | LOW | The manufacturer provided the study products |
| Chapple et al. | 2012 | LOW | - | LOW | LOW | LOW | LOW | LOW | The manufacturer of the drug supported the study |
| Gilowski et al. | 2012 | LOW | - | LOW | LOW | LOW | LOW | LOW | None |
| Parvu et al. | 2013 | SOME CONCERNS | No information about randomization method | SOME CONCERNS | LOW | LOW | LOW | LOW | The manufacturer of the drug provided a gift grant |
| Teughels et al. | 2013 | LOW | - | LOW | LOW | LOW | LOW | LOW | The manufacturer provided a research grant |
| Deore et al. | 2014 | LOW | - | LOW | LOW | LOW | LOW | LOW | Not declared |
| Singh et al. | 2014 | SOME CONCERNS | No information about allocation; Absence of placebo | SOME CONCERNS | SOME CONCERNS | LOW | LOW | LOW | None |
| Elwakeel et al. | 2015 | SOME CONCERNS | Allocation concealment not clear; Number of droputs | SOME CONCERNS | LOW | LOW | LOW | LOW | None |
| Ince et al. | 2015 | LOW | - | LOW | LOW | LOW | LOW | LOW | The manufacturer of the probiotic supported the study providing tablets |
| Laleman et al. | 2015 | LOW | - | LOW | LOW | LOW | LOW | LOW | The manufacturer of the probiotic supported the study providing tablets |
| Tekce et al. | 2015 | LOW | - | LOW | LOW | LOW | LOW | LOW | The manufacturer provided the study products |
| Morales et al. | 2016 | LOW | - | LOW | LOW | LOW | LOW | LOW | The manufacturer of the probiotic supported the study |
| Alyousef et al. | 2017 | LOW | - | LOW | LOW | HIGH | LOW | LOW | None |
| Chitsazi et al. | 2017 | SOME CONCERNS | Allocation concealment not clear; Absence of placebo | SOME CONCERNS | SOME CONCERNS | LOW | LOW | LOW | None |
| Keskiner et al. | 2017 | SOME CONCERNS | No information about allocation / randomization | SOME CONCERNS | LOW | LOW | LOW | LOW | The manufacturer of the probiotic supported the study providing tablets |
| Umrania et al. | 2017 | SOME CONCERNS | Absence of placebo | LOW | SOME CONCERNS | LOW | LOW | LOW | None |
| Invernici et al. | 2018 | LOW | - | LOW | LOW | LOW | LOW | LOW | The manufacturer of the probiotic supported the study providing tablets |
| Morales et al. | 2018 | LOW | - | LOW | LOW | LOW | LOW | LOW | None |
| Surapaneni et al. | 2018 | SOME CONCERNS | No information about allocation / randomization; Absence of placebo | SOME CONCERNS | SOME CONCERNS | LOW | LOW | LOW | None |
| El-Sharkawy et al. | 2019 | LOW | No ITT analysis | LOW | LOW | LOW | LOW | LOW | None |
| Hong et al. | 2019 | LOW | No ITT analysis | LOW | LOW | LOW | LOW | LOW | The manufacturer of the probiotic supported the study providing the drug |
| Pelekos et al. | 2019 | LOW | - | LOW | LOW | LOW | LOW | LOW | The manufacturer of the drug supported the study providing lozenges |
| Rampally et al. | 2019 | SOME CONCERNS | No information about allocation / randomization; Absence of placebo | SOME CONCERNS | SOME CONCERNS | LOW | LOW | LOW | None |
| Soares et al. | 2019 | SOME CONCERNS | No information about allocation | SOME CONCERNS | LOW | LOW | LOW | LOW | None |
| Sravya et al. | 2019 | SOME CONCERNS | No information about allocation / randomization; Absence of placebo | SOME CONCERNS | SOME CONCERNS | LOW | LOW | LOW | None |
| Theodoro et al. | 2019 | LOW | - | LOW | LOW | LOW | LOW | LOW | None |
| dos Santos et al. | 2020 | LOW | - | LOW | LOW | LOW | LOW | LOW | None |
| Tinto et al. | 2020 | LOW | - | LOW | LOW | LOW | LOW | LOW | None |
| Vohra et al. | 2020 | SOME CONCERNS | No information about allocation / randomization; Absence of placebo | SOME CONCERNS | SOME CONCERNS | LOW | LOW | LOW | None |

ITT = intention to treat

***Appendix 4***

Outcomes of the included studies.

| **AUTHORS and YEAR** | Follow-up (months) | Sites | Negative control group | | | | Test group 1 | | | | Test group 2 | | | |
| --- | --- | --- | --- | --- | --- | --- | --- | --- | --- | --- | --- | --- | --- | --- |
|  |  |  | ∆ PD Negative control group | ∆ CAL Negative control group | ∆ Plaque  Negative control group | ∆ Bleeding  Negative control group | ∆ PD  Test group 1 | ∆ CAL  Test group 1 | ∆ Plaque  Test group 1 | ∆ Bleeding  Test group 1 | ∆ PD Test group 2 | ∆ CAL  Test group 2 | ∆ Plaque  Test group 2 | ∆ Bleeding  Test group 2 |
| Rocha et al., 2004 | 6 | All sites: | 0.4 ± 0.4 | 0.5 ± 0.8 | 40 ± 10 ° | 20 ± 6 • | 0.8 ± 0.3 | 0.99 ± 0.8 | 30 ± 10 ° | 33 ± 10 • | - | - | - | - |
| Lee et al., 2004 | 3 | All sites: | 1.04 ± 0.05 | 0.5 ± 0.06 | - | - | 1.52 ± 0.05 | 0.96 ± 0.06 | - | - | - | - | - | - |
|  | 6 | All sites: | 1.61 ± 0.05 | 0.63 ± 0.06 | - | - | 1.61 ± 0.05 | 1.42 ± 0.05 | - | - | - | - | - | - |
|  | 9 | All sites: | 1.19 ± 0.06 | 0.8 ± 0.05 | - | - | 1.63 ± 0.07 | 1.56 ± 0.06 | - | - |  | - | - | - |
| Preshaw et al., 2004 | 3 | PD 4-6:  PD ≥ 7: | 0.91 ± 0.06  1.76 ± 0.13 | 0.88 ± 0.06  1.59 ± 0.14 | - | - | 1.09 ± 0.05  1.88 ± 0.1 | 1.03 ± 0.05  1.69 ± 0.11 | - | - | - | - | - | - |
|  | 6 | PD 4-6:  PD ≥ 7: | 1.03 ± 0.06  1.52 ± 0.16 | 1.09 ± 0.05  1.44 ± 0.17 | - | - | 1.19 ± 0.05  2.15 ± 0.12 | 1.19 ± 0.05  1.96 ± 0.13 | - | - | - | - | - | - |
|  | 9 | PD 4-6:  PD ≥ 7: | 0.96 ± 0.06  1.77 ± 0.13 | 0.94 ± 0.05  1.6 ± 0.15 | - | - | 1.29 ± 0.05  2.31 ± 0.12 | 1.27 ± 0.05  2.09 ± 0.13 | - | - | - | - | - | - |
| Mohammad et al., 2005 | 3 | PD 4-5:  PD ≥ 6: | 0.63 ± 0.1  0.75 ± 0.32 | 0.33 ± 0.25  0.2 ± 0.59 | - | 13.7 ± 41.9•* (all) | 1.64 ± 0.1  2.62 ± 0.3 | 1.27 ± 0.25  1.46 ± 0.54 | - | 46.5 ± 28.9•* (all) | - | - | - | - |
|  | 6 | PD 4-5:  PD ≥ 6: | 0.67 ± 0.14  0.81 ± 0.29 | 0.33 ± 0.28  0.23 ± 0.56 | - | -5.9 ± 37.5•* (all) | 1.45 ± 0.14  3.06 ± 0.27 | 1.98 ± 0.28  3.17 ± 0.51 | - | 52 ± 29.7•* (all) | - | - | - | - |
|  | 9 | PD 4-5:  PD ≥ 6: | 0.63 ± 0.11  0.98 ± 0.31 | 0.02 ± 0.21  0.25 ± 0.64 | - | -18.1 ± 36.7•* (all) | 1.57 ± 0.11  3.22 ± 0.29 | 2.14 ± 0.21  3.18 ± 0.59 | - | 60.4 ± 27.9•* (all) | - | - | - | - |
| Gorska & Nedzi-Gora, 2006 | 3 | All sites: | 0.08 ± 0.03 * | 0.04 ± 0.05 * | 23.51 ± 2.16 °* | 12.05 ± 1.1 •* | 0.29 ± 0.03 * | 0.33 ± 0.03 * | 30.19 ± 2.77 °* | 27.79 ± 2.55 •* | - | - | - | - |
| Emingil et al., 2006 | 3 | All sites: | 1.29 ± 0.27 * | 0.46 ± 0.1 * | 2.11 ± 0.44 §* | 0.89 ± 0.19 #* | 1.38 ± 0.29 * | 0.58 ± 0.12 * | 1.88 ± 0.39 §* | 1.06 ± 0.22 #* | - | - | - | - |
|  | 6 | All sites: | 1.3 ± 0.27 * | 0.33 ± 0.07 * | 2.23 ± 0.46 §* | 0.87 ± 0.18 #* | 1.5 ± 0.31 * | 0.55 ± 0.11 * | 1.87 ± 0.39 §* | 0.99 ± 0.21 #* | - | - | - | - |
|  | 9 | All sites: | 1.27 ± 0.26 * | 0.35 ± 0.07 * | 2.29 ± 0.48 §* | 0.92 ± 0.19 #* | 1.57 ± 0.33 * | 0.62 ± 0.13 * | 2.87 ± 0.6 §* | 1.12 ± 0.23 #* | - | - | - | - |
|  | 12 | All sites: | 1,26 ± 0.26 * | 0.35 ± 0.07 * | 2.31 ± 0.48 §* | 0.97 ± 0.2 #* | 1.6 ± 0.33 * | 0.55 ± 0.11 * | 2.43 ± 0.51 §* | 1.12 ± 0.23 #* | - | - | - | - |
| Needleman et al., 2007 | 6 | PD ≥ 5: | 0.98 ± 2.58 | 0.4 ± 1.72 | 19.3 ± 103.25 ° | 27.2 ± 81.39 • | 1.4 ± 2.86 | 0.65 ± 2.04 | 21.7 ± 60.1 ° | 21.8 ± 59.7 • | - | - | - | - |
| Preshaw et al., 2008 | 3 | PD 4-6:  PD ≥ 7: | 1.28 ± 0.08  2.1 ± 0.24 | 1.33 ± 0.1  2.07 ± 0.27 | - | 1.1± 0.1†  1 ± 0.2† | 1.49 ± 0.08  2.56 ± 0.24 | 1.48 ± 0.1  2.38 ± 0.27 | - | 1.19 ± 0.13†  1.2 ± 0,16† | - | - | - | - |
|  | 6 | PD 4-6:  PD ≥ 7: | 1.4 ± 0.12  2.25 ± 0.29 | 1.5 ± 0.16  2.24 ± 0.32 | - | 1.2 ± 0.1†  1.2 ± 0.1† | 1.61 ± 0.12  2.84 ± 0.29 | 1.67 ± 0.16  2.71 ± 0.32 | - | 1.26 ± 0.13†  1.31 ± 0.13† | - | - | - | - |
|  | 9 | PD 4-6:  PD ≥ 7: | 1.41 ± 0.13  2.25 ± 0.24 | 1.49 ± 0.17  2.2 ± 0.31 | - | 1.2 ± 0.1†  1.2 ± 0.1† | 1.63 ± 0.13  2.74 ± 0.24 | 1.66 ± 0.17  2.62 ± 0.31 | - | 1.27 ± 0.12†  1.31 ± 0.14† | - | - | - | - |
| Alec Yen et al., 2008 | 3 | PD 1-3:  PD 4-6:  PD ≥ 7: | 0.11 ± 0.59  0.98 ± 0.9  1.89 ± 1.6 | 0.05 ± 0.78  0.83 ± 1.1  1.46 ± 1.87 | - | - | 0.12 ± 0.55  1.14 ± 0.92  3.27 ± 1.56 | 0.13 ± 0.71  1.06 ± 1.01  3.04 ± 1.61 | - | - | - | - | - | - |
|  | 6 | PD 1-3:  PD 4-6:  PD ≥ 7: | 0.13 ± 0.6  1.16 ± 1.06  2.1 ± 2.09 | 0.05 ± 0.77  1 ± 1.16  1.52 ± 2.22 | - | - | 0.13 ± 0.56  1.23 ± 0.93  3.31 ± 1.51 | 0.12 ± 0.71  1.13 ± 1  3.06 ± 1.68 | - | - | - | - | - | - |
|  | 9 | PD 1-3:  PD 4-6:  PD ≥ 7: | 0.12 ± 0.71  1.2 ± 1.08  2.2± 2.14 | 0.04 ± 0.91  1.03 ± 1.21  1.41 ± 2.46 | - | - | 0.1 ± 0.58  1.2 ± 0.99  3.33 ± 1.8 | 0.08 ± 0.73  1.12 ± 1.04  3.05 ± 1.73 | - | - | - | - | - | - |
|  | 12 | PD 1-3:  PD 4-6:  PD ≥ 7: | 0.09 ± 0.71  0.99 ± 1.37  2.06 ± 2.88 | 0.04 ± 0.9  0.8 ± 2.18  1.43 ± 2.9 | - | - | 0.09 ± 0.66  1.25 ± 0.99  3.74 ± 1.83 | 0.09 ± 0.08  1.22 ± 1.04  3.74 ± 1.83 | - | - | - | - | - | - |
| Graziani et al., 2009 | 3 | All sites:  PD 1-3:  PD 4-6:  PD ≥ 7: | 0.7 ± 0.55  0 ± 0.27  1.7 ± 0.82  3 ± 1.65 | 0.5 ± 0.69  -0.1 ± 0.55  1.2 ± 0.96  2.8 ± 1.65 | - | 18.3 ± 26.5 • (all) | 0.8 ± 0.65  0.1 ± 0.39  1.7 ± 0.78  2.7 ± 1.82 | 0.6 ± 0.65  0 ± 0.39  1.3 ± 0.78  2.2 ± 1.82 | - | 15.4 ± 23.9 • (all) | - | - | - | - |
|  | 6 | All sites:  PD 1-3:  PD 4-6:  PD ≥ 7: | 0.7 ± 0.52  0 ± 0.03  1.8 ± 0.65  3.2 ± 1.56 | 0.6 ± 0.78  -0.1 ± 0.65  1.3 ± 1.95  3.1 ± 1.56 | - | 14.9 ± 25.6 • (all) | 0.7 ± 0.13  0 ± 0.13  1.7 ± 0.76  3 ± 1.91 | 0.5 ± 0.77  -0.2 ± 0.77  1.1 ± 0.89  2.6 ± 2.04 | - | 11.4 ± 21.3 • (all) | - | - | - | - |
| Sulaiman & Shehadeh, 2010 | 3 | All sites: | 0.55 ± 0.09 * | 0.56 ± 0.09 * | 0 ± 0 §* | 0.47 ± 0.08 #*  22.25 ± 3.66 •* | 0.55 ± 0.09 * | 0.54 ± 0.09 * | 0.1 ± 0.02 §* | 0.34 ± 0.06 #*  19.13 ± 3.15 •* | - | - | - | - |
| Gilowski et al., 2012 | 3 |  | - | - | - | - | - | - | - | - | - | - | - | - |
| Chapple et al., 2012 | 3 | All sites: | 0.83 ± 0.11 * | 0.25 ± 1.05 * | - | 15 ± 2.53 •* | 0.88 ± 0.11 * | 0.4 ± 0.11 * | - | 22.1 ± 3.74 •* | 0.91 ± 0.12 * | 0.29 ± 0.07 * | - | 17.1 ± 2.9 •* |
|  | 6 | All sites: | 1.02 ± 0.13 * | 0.25 ± 1.05 * | - | 15.9 ± 2.71 •* | 0.93 ± 0.12 * | 0.2 ± 0.06 * | - | 23.9 ± 4.09 •* | 1.08 ± 0.14 * | 0.37 ± 0.09 * | - | 19.4 ± 2.9 •* |
|  | 9 | All sites: | 1.04 ± 0.14 * | 0.15 ± 1.05 * | - | 15.3 ± 2.61 •* | 1 ± 0.12 * | 0.2 ± 0.06 * | - | 23.8 ± 4.07 •* | 1.09 ± 0.14 * | 0.37 ± 0.09 * | - | 16.9 ± 2.89 •* |
| Teughels et al., 2013 | 3 | All sites:  PD 4-6:  PD ≥ 7: | 1.39 ± 0.15  1.72 ± 0.17  2.25 ± 0.27 | 0.76 ± 0.36  1.01 ± 0.59  0.68 ± 0.85 | - | 50.95 ± 15.03 • | 1.41 ± 0.25  1.84 ± 0.22  2.88 ± 0.35 | 0.99 ± 0.22  1.42 ± 0.27  1.47 ± 0.71 | - | 52.02 ± 15.34 • | - | - | - | - |
| Parvu et al., 2013 | 3 | All sites: | 2.76 ± 0.14 * | 1.59 ± 0.18 * | - | 21.4 ± 1.6 •* | 2.65 ± 0.14 * | 1.9 ± 0.15 * | - | 41.7 ± 2.48 •* | - | - | - | - |
| Deore et al., 2014 | 3 | All sites: | 1.28 ± 0.33 * | 1.48 ± 0.38 * | 0.85 ± 0.22 §* | 0.8 ± 0.2 †*  0.61 ± 0.16 #* | 1.9 ± 0.48 * | 2.8 ± 0.71 * | 0.88 ± 0.22 §* | 1.11 ± 0.22 †*  0.81 ± 0.21 #* | - | - | - | - |
| Singh et al., 2014 | 3 | All sites: | 1.19 (0.40, 2.05)  (median; MIN, MAX) | 1.13 (0.23, 2.41)  (median; MIN, MAX) | 1.02 (0.03, 1.88) §  (median; MIN, MAX) | 47.80 (38.12, 58.67) •  (median; MIN, MAX) | 1.68 (0.27, 3.44) (median; MIN, MAX) | 1.76 (0.04, 3.57) (median; MIN, MAX) | 1.41 (-0.06, 2.17) §  (median; MIN, MAX) | 60.76 (46.30, 73.01) •  (median; MIN, MAX) | - | - | - | - |
| Laleman et al., 2015 | 3 | All sites:  PD 4-6:  PD ≥ 7: | 1.34 ± 0.33  1.54 ± 0.35  2.41 ± 0.69 | 0.7 ± 0.3  0.92 ± 0.22  1.39 ± 0.76 | - | 57 ± 9• (all) | 1.35 ± 0.3  1.62 ± 0.35  2.37 ± 0.83 | 0.76 ± 0.2  1.03 ± 0.21  1.32 ± 0.68 | 41.1 ± 10.97 °* | 60 ± 12• (all) | - | - | - | - |
|  | 6 | All sites:  PD 4-6:  PD ≥ 7: | 1.62 ± 0.41  1.82 ± 0.42  3.43 ± 0.84 | 0.75 ± 0.37  0.98 ± 0.3  1.82 ± 0.65 | - | 48 ± 19• (all) | 1.52 ± 0.38  1.78 ± 0.38  3.2 ± 0.99 | 0.71 ± 0.28  0.98 ± 0.25  1.65 ± 0.54 | 47.3 ± 12.63 °* | 47 ± 17• (all) | - | - | - | - |
| Tekce et al., 2015 | 3 | All sites: | 0.85 ± 0.32 | 0.79 ± 0.32 | 1.16 ± 0.18 §* | 0.59 ± 0.09 #*  66.8 ± 10.5 • | 1.44 ± 0.33 | 1.18 ± 0.36 | 1.69 ± 0.27 §* | 1.36 ± 0.21 #*  72.25 ± 11.4 • | - | - | - | - |
|  | 6 | All sites: | 0.7 ± 0.24 | 0.66 ± 0.22 | 1.07 ± 0.17 §* | 0.58 ± 0.09 #*  68.7 ± 10.8 • | 1.77 ± 0.69 | 0.67 ± 0.24 | 1.66 ± 0.26 §* | 1.43 ± 0.23 #*  76.6 ± 12.8 • | - | - | - | - |
|  | 12 | All sites: | 0.57 ± 0.24 | 0.53 ± 0.24 | 0.91 ± 0.14 §* | 0.46 ± 0.07 #*  69.6 ± 11 • | 1.74 ± 0.62 | 1.39 ± 0.26 | 1.49 ± 0.23 §* | 1.32 ± 0.21 #*  77.85 ± 12.28• | - | - | - | - |
| Ince et al., 2015 | 3 | All sites: | 1.2 ± 0.32 | 0.59 ± 0.32 | 1.04 ± 0.2 §* | 0.48 ± 0.14 #*  66.25 ± 10.9 • | 1.6 ± 0.39 | 1.08 ± 0.36 | 1.6 ± 0.26 §* | 1.46 ± 0.43 #*  71.43 ± 11.75 • | - | - | - | - |
|  | 6 | All sites: | 0.7 ± 0.24 | 0.46 ± 0.22 | 0.97 ± 0.21 §* | 0.51 ± 0.15 #*  68.78 ± 11.31 • | 1.81 ± 0.32 | 1.27 ± 0.24 | 1.57 ±0.26 §* | 1.51 ± 0.45 #*  75.97 ± 12.5 • | - | - | - | - |
|  | 12 | All sites: | 0.55 ± 0.26 | 0.43 ± 0.24 | 0.8 ± 0.24 §* | 0.38 ± 0.11 #*  69.65 ± 11.5 • | 1.7 ± 0.31 | 1.39 ± 0.26 | 1.49 ± 0.25 §* | 1.42 ± 0.42 #*  - | - | - | - | - |
| Elwakeel et al., 2015 | 3 | All sites: | 1.3 ± 0.36 * | 1.5 ± 0.42 * | - | 0.45 ± 0.13 #* | 2.2 ± 0.61 * | 1.9 ± 0.53 * | - | 1.45 ± 0.4 #* | - | - | - | - |
|  | 6 | All sites: | 2.05 ± 0.57 * | 2.4 ± 0.67 * | - | 1.15 ± 0.32 #* | 2.9 ± 0.81 * | 3.05 ± 0.85 * | - | 1.95 ± 0.54 #* | - | - | - | - |
| Morales et al., 2016 | 3 | All sites: | 0.4 ± 0.4 | 0.7 ± 1.3 | 25.6 ± 14.4 ° | 10.3 ± 14.6 • | 0.5 ± 0.2 | 0.05 ± 0.1 | 32 ± 16 ° | 12.9 ± 15.9 • | - | - | - | - |
|  | 6 | All sites: | 0.4 ± 0.4 | 0.7 ± 1 | 23 ± 13.5 ° | 5.9 ± 14.9 • | 0.6 ± 0.3 | 0.3 ± 0.6 | 32.7 ± 11.4 ° | 11.3 ± 14.4 • | - | - | - | - |
|  | 9 | All sites: | 0.4 ± 0.4 | 0.09 ± 0.8 | 20.9 ± 14.3 ° | 9.8 ± 17.9 • | 0.5 ± 0.3 | 0.07 ± 0.5 | 33.5 ± 12.2 ° | 12.8 ± 19.7 • | - | - | - | - |
|  | 12 | All sites: | 0.4 ± 0.4 | 0.09 ± 0.8 | 16.6 ± 17 ° | 8.3 ± 18.4 • | 0.6 ± 0.3 | 0.07 ± 0.5 | 30 ± 11.5 ° | 11.7 ± 20.3 • | - | - | - | - |
| Alyousef et al., 2017 | 3 | All sites: | 1.96 ± 0.39 * | - | 1.16 ± 0.36 §* | 0.6 ± 0.5 †* | 2.31 ± 0.55 * | - | 1.5 ± 0.52 §* | 1.13 ± 0.33 †* | - | - | - | - |
| Umrania et al., 2017 | 3 | All sites: | 0.29 ± 0.08 * | 0.33 ± 0.09 | 1.86 ± 0.29 §* | 1.85 ± 0.29 #* | 0.34 ± 0.1 * | 0.42 ± 0.12 | 1.98 ± 0.31 §* | 1.94 ± 0.31 #* | - | - | - | - |
| Chitsazi et al., 2017 | 3 | All sites: | 1.17 ± 0.41 | 1.09 ± 0.53 | - | 0.58 ± 0.09 #* | 1.85 ± 0.7 | 2.06 ± 0.47 | - | 0.54 ± 0.12 # | 2.02 ± 0.41 | 2.18 ± 0.55 | - | 0.63 ± 0.11 # |
|  | 6 | All sites: | 1.48 ± 0.4 | 1.67 ± 0.61 | - | 0.46 ± 0.07 #* | 2.87 ± 0.69 | 3.07 ± 0.55 | - | 0.83 ± 0.21 # | 3.35 ± 0.64 | 3.3 ± 0.66 | - | 0.92 ± 0.22 # |
| Keskiner et al., 2017 | 3 | All sites: | - | - | - | - | - | - | - | - | - | - | - | - |
|  | 6 | All sites: | - | - | - | - | - | - | - | - | - | - | - | - |
| El-Sharkawy et al., 2018 | 3 | All sites: | 1.2 ± 0.2 | 1.3 ± 0.3 | 1.31 ± 0.18 §* | 1.54 ± 0.22 #*  43 ± 6 •* | 1.9 ± 0.3 | 2.1 ± 0.5 | 1.51 ± 0.21 §* | 1.41 ± 0.2 #*  52 ± 7.2 •* | - | - | - | - |
|  | 6 | All sites: | 1.3 ± 0.3 | 1.4 ± 0.4 | 1.28 ± 0.18 §* | 1.52 ± 0.21 #*  41 ± 5.7 •* | 2 ± 0.4 | 2.2 ± 0.4 | 1.54 ± 0.21 §* | 1.46 ± 0.2 #*  51 ± 7.1 •* | - | - | - | - |
| Invernici et al., 2018 | 3 | All sites:  PD 4-6:  PD ≥ 7: | 0.25 ± 0.22  0.94 ± 0.42  2.62 ± 0.88 | 0.18 ± 0.23  0.76 ± 0.41  2.07 ± 0.97 | -0.47 ± 12.07 ° (all sites) | 4.29 ± 18.61• (all) | 0.52 ± 0.32  1.28 ± 0.41  3.52 ± 1.19 | 0.49 ± 0.37  1.15 ± 0.45  3.45 ± 1.21 | 2.2 ± 12.3 ° (all sites) | 12 ± 15.49• (all) | - | - | - | - |
| Morales et al., 2018 | 3 | All sites: | 0.7 ± 0.4 * | 0.6 ± 1.3 * | 23.7 ± 14.4 °* | 11.8 ± 14.6 • | 0.6 ± 0.2 * | 0.4 ± 0.1 * | 29.8 ± 16 °* | 24.6 ± 15.9 • | - | - | - | - |
|  | 6 | All sites: | 0.7 ± 0.4 * | 0.6 ± 1 * | 27.4 ± 13.5 °* | 11 ± 14.9 • | 0.4 ± 0.3 * | 0.5 ± 0.6 * | 29.3 ± 11.4 °* | 24.1 ± 14.4 • | - | - | - | - |
|  | 9 | All sites: | 0.7 ± 0.4 * | 0.4 ± 0.8 * | 28.7 ± 14.3 °* | 6.6 ± 17.9 • | 0.5 ± 0.3 * | 0.4 ± 0.5 * | 26.4 ± 12.2 °* | 21.2 ± 19.7 • | - | - | - | - |
| Surapaneni et al., 2018 | 3 | All sites: | 3.4 ± 0.47 * | 3.4 ± 0.47 * | - | 1.3 ± 0.21 #* | 4.3 ± 0.59 * | 4.4 ± 0.69 * | - | 1.6 ± 0.25 #* | - | - | - | - |
| Hong et al., 2019 | 3 | All sites: | 0.1 ± 0.36 * | 0.02 ± 0.46 | 0.02 ± 0.39 § | 0.1 ± 0.44 # | 0.11 ± 0.34 * | 0.03 ± 0.48 | 0.18 ± 0.52 § | 0.24 ± 0.38 # | - | - | - | - |
| Rampally et al., 2019 | 3 | All sites: | 2 ± 0.33 * | 2 ± 0.33 * | - | 0.82 ± 0.14 #* | 2 ± 0.33 * | 2 ± 0.33 * | - | 0.8 ± 0.13 #* | 2 ± 0.33 * | 2 ± 0.33 * | - | 0.77 ± 0.13 #* |
| Pelekos et al., 2019 | 3 | All sites: | 0.6 ± 0.5 | 0.2 ± 0.2 | 23.1 ± 18.3 ° | 26.8 ± 20.2 • | 0.4 ± 0.2 | 0.2 ± 0.2 | 15.4 ± 14.5 ° | 22.1 ± 26.3 • | - | - | - | - |
|  | 6 | All sites: | 0.7 ± 0.5 | 0.3 ± 0.2 | 29.1 ± 18.5 ° | 32.4 ± 19 • | 0.5 ± 0.3 | 0.2 ± 0.2 | 19.6 ± 15.9 ° | 29.9 ± 15.2 • | - | - | - | - |
| Theodoro et al., 2019 | 3 | All sites:  PD 5-6:  PD ≥ 7: | 0.15 ± 0.04 *  0.01 ± 0.13  1.67 ± 3.24 | 0.06 ± 0.1 *  -0.4 ± 1.57  0.15 ± 1.59 | - | 8.97 ± 2.63 •* (all) | 0.25 ± 0.07 *  0.36 ± 1.38  2.8 ± 3.75 | 0.43 ± 0.13 *  0.08 ± 0.16  0.76 ± 2.19 | - | 22.23 ± 6.53 •* (all) | - | - | - | - |
| Soares et al., 2019 | 3 | All sites: | 1.1 ± 0.28 * | 0 ± 0 * | 9 ± 2.28 °* | 14 ± 3.56 •* | 2.1 ± 0.53 | -0.3 ± 0.08 * | 14 ± 3.56 °* | 27 ± 6.85 •* | - | - | - | - |
| Vohra et al, 2020 | 3 | All sites: | 2.7 ± 0.68 * | 0.1 ± 0.03 * | 26.5 ± 6.68 °* | 39 ± 9.82 •* | 3.6 ± 0.89 * | 0.3 ± 0.07 * | 37.8 ± 9.39 °* | 42.6 ± 10.58 •* | - | - | - | - |
|  | 6 | All sites: | 2.75 ± 0.79 * | 0.2 ± 0.05 * | 23.1 ± 5.82 °* | 38.2 ± 9.62 •* | 2.4 ± 0.6 * | 0.2 ± 0.05 * | 25.9 ± 6.43 °* | 35.1 ± 8.72 •* | - | - | - | - |
| dos Santos et al., 2020 | 3 | All sites: | 0.4 ± 0.3 * | 0.3 ± 0.4 * | 18.9 ± 25.2 °* | 16.4 ± 17.9 •* (all) | 0.5 ± 0.3 * | 0.4 ± 0.6 * | 15.3 ± 21.1 °* | 15.7 ± 14.9 •* (all) | - | - | - | - |
|  | 6 | All sites:  PD 5-6:  PD ≥ 7: | 0.4 ± 0.3 *  1.1 ± 0.2  2 ± 0.2 | 0.2 ± 0.4 *  0.8 ± 0.2  1.6 ± 0.3 | 14.1 ± 25.2 °* | 14.1 ± 17.9 •* (all) | 0.4 ± 0.3 *  1.2 ± 0.2  1.9 ± 0.2 | 0.5 ± 0.6 *  1.3 ± 0.2  2.6 ± 0.3 | 20.5 ± 21.1 °* | 17.5 ± 14.9 •* (all) | - | - | - | - |
| Tinto et al., 2020 | 6 | All sites:  PD 4-5:  PD ≥ 6: | 0.73 ± 0.19 *  1.04 ± 0.69  2.11 ± 0.96 | - | - | - | 1.27 ± 0.33 *  1.86 ± 0.81  3.33 ± 1.43 | - | - | - | - | - | - | - |

Results are reported as mean ± standard deviations. Symbols: * = the value was not reported directly in the original paper but it was possible to calculate it; † = sulcus bleed; # = GI; • = BOP%; § = PI; ° = PI%. Missing results where not reported nor calculable from the original paper.

***Appendix 5***

| **Summary of findings:** | | | | | | |
| --- | --- | --- | --- | --- | --- | --- |
| **NSPT + Omega 3 compared to NSPT (+ Placebo) for periodontitis** | | | | | | |
| **Patient or population**: periodontitis  **Setting**:  **Intervention**: NSPT + Omega 3  **Comparison**: NSPT (+ Placebo) | | | | | | |
| Outcomes | **Anticipated absolute effects^*^** (95% CI) | | Relative effect (95% CI) | № of participants  (studies) | Certainty of the evidence (GRADE) | Comments |
|  | **Risk with NSPT (+ Placebo)** | **Risk with NSPT + Omega 3** |  |  |  |  |
| PD reduction (3 months) | The mean PD reduction (3 months) was **1.13** mm | MD **0.21 mm higher** (0.12 lower to 0.55 higher) | - | 126 (3 RCTs) | ⨁◯◯◯ VERY LOW ^a,b,c,d^ |  |
| CAL gain (3 months) | The mean CAL gain (3 months) was **1.25** mm | MD **0.46 mm higher** (0.19 lower to 1.11 higher) | - | 126 (3 RCTs) | ⨁◯◯◯ VERY LOW ^a,b,c,d^ |  |
| GI reduction (3 months) | The mean GI reduction (3 months) was **1.03** | MD **0.08 higher** (0.09 lower to 0.25 higher) | - | 126 (3 RCTs) | ⨁◯◯◯ VERY LOW ^a,b,c,d^ |  |
| ***The risk in the intervention group** (and its 95% confidence interval) is based on the assumed risk in the comparison group and the **relative effect** of the intervention (and its 95% CI).   **CI:** Confidence interval; **MD:** Mean difference | | | | | | |
| **GRADE Working Group grades of evidence** **High certainty:** We are very confident that the true effect lies close to that of the estimate of the effect **Moderate certainty:** We are moderately confident in the effect estimate: The true effect is likely to be close to the estimate of the effect, but there is a possibility that it is substantially different **Low certainty:** Our confidence in the effect estimate is limited: The true effect may be substantially different from the estimate of the effect **Very low certainty:** We have very little confidence in the effect estimate: The true effect is likely to be substantially different from the estimate of effect | | | | | | |

#### Explanations

a. Downgraded due to risk of bias

b. Downgraded due to heterogeneity

c. Downgraded due to differences in populations and interventions

d. Downgraded due to 95% CI boundaries

| **Summary of findings:** | | | | | | |
| --- | --- | --- | --- | --- | --- | --- |
| **NSPT + Vitamin complex compared to NSPT (+ Placebo) for periodontitis** | | | | | | |
| **Patient or population**: periodontitis  **Setting**:  **Intervention**: NSPT + Vitamin complex  **Comparison**: NSPT (+ Placebo) | | | | | | |
| Outcomes | **Anticipated absolute effects^*^** (95% CI) | | Relative effect (95% CI) | № of participants  (studies) | Certainty of the evidence (GRADE) | Comments |
|  | **Risk with NSPT (+ Placebo)** | **Risk with NSPT + Vitamin complex** |  |  |  |  |
| PD reduction (3 months) | The mean PD reduction (3 months) was **0.37** mm | MD **0.02 mm higher** (0.02 lower to 0.07 higher) | - | 163 (3 RCTs) | ⨁⨁◯◯ LOW ^a,b^ |  |
| CAL gain (3 months) | The mean CAL gain (3 months) was **0.18** mm | MD **0.01 mm lower** (0.07 lower to 0.05 higher) | - | 163 (3 RCTs) | ⨁⨁◯◯ LOW ^a,b^ |  |
| ***The risk in the intervention group** (and its 95% confidence interval) is based on the assumed risk in the comparison group and the **relative effect** of the intervention (and its 95% CI).   **CI:** Confidence interval; **MD:** Mean difference | | | | | | |
| **GRADE Working Group grades of evidence** **High certainty:** We are very confident that the true effect lies close to that of the estimate of the effect **Moderate certainty:** We are moderately confident in the effect estimate: The true effect is likely to be close to the estimate of the effect, but there is a possibility that it is substantially different **Low certainty:** Our confidence in the effect estimate is limited: The true effect may be substantially different from the estimate of the effect **Very low certainty:** We have very little confidence in the effect estimate: The true effect is likely to be substantially different from the estimate of effect | | | | | | |

#### Explanations

a. Downgraded due to risk of bias

b. Downgraded due to 95% CI boundaries

| **Summary of findings:** | | | | | | | | | | | |
| --- | --- | --- | --- | --- | --- | --- | --- | --- | --- | --- | --- |
| **NSPT + Probiotics compared to NSPT (+ Placebo) [All sites] for periodontitis** | | | | | | | | | | | |
| **Patient or population**: periodontitis  **Setting**:  **Intervention**: NSPT + Probiotics  **Comparison**: NSPT (+ Placebo) [All sites] | | | | | | | | | | | |
| Outcomes | | **Anticipated absolute effects^*^** (95% CI) | | | | Relative effect (95% CI) | | № of participants  (studies) | | Certainty of the evidence (GRADE) | Comments |
|  |  | **Risk with NSPT (+ Placebo) [All sites]** | | **Risk with NSPT + Probiotics** | |  |  |  |  |  |  |
| PD reduction (3 months) | The mean PD reduction (3 months) was **1.07** mm | | MD **0.3 mm higher** (0.11 higher to 0.48 higher) | | - | | 441 (11 RCTs) | | ⨁⨁◯◯ LOW ^a,b^ | |  |
| PD reduction (6 months) | The mean PD reduction (6 months) was **1.29** mm | | MD **0.2 mm higher** (0.27 lower to 0.68 higher) | | - | | 282 (7 RCTs) | | ⨁⨁◯◯ LOW ^a,b^ | |  |
| PD reduction (12 months) | The mean PD reduction (12 months) was **0.52** mm | | MD **0.84 mm higher** (0.22 higher to 1.46 higher) | | - | | 98 (3 RCTs) | | ⨁⨁◯◯ LOW ^a,b^ | |  |
| CAL gain (3 months) | The mean CAL gain (3 months) was **0.38** mm | | MD **0.21 mm higher** (0.11 higher to 0.31 higher) | | - | | 441 (11 RCTs) | | ⨁⨁◯◯ LOW ^a,b^ | |  |
| CAL gain (6 months) | The mean CAL gain (6 months) was **0.50** mm | | MD **0.21 mm higher** (0.15 lower to 0.56 higher) | | - | | 282 (7 RCTs) | | ⨁⨁◯◯ LOW ^a,b^ | |  |
| CAL gain (12 months) | The mean CAL gain (12 months) was **0.37** mm | | MD **0.7 mm higher** (0.36 higher to 1.04 higher) | | - | | 98 (3 RCTs) | | ⨁⨁◯◯ LOW ^a,b^ | |  |
| BOP% reduction (3 months) | | The mean BOP% reduction (3 months) was **32.71** % | | MD **6.85 % higher** (3.36 higher to 10.34 higher) | | - | | 441 (11 RCTs) | | ⨁⨁◯◯ LOW ^a,b^ |  |
| BOP% reduction (6 months) | | The mean BOP% reduction (6 months) was **40.56** % | | MD **3.5 % higher** (1.46 lower to 8.47 higher) | | - | | 282 (7 RCTs) | | ⨁◯◯◯ VERY LOW ^a,b,c^ |  |
| BOP% reduction (12 months) | | The mean BOP% reduction (12 months) was **52.10** % | | MD **7.41 % higher** (2.34 higher to 12.49 higher) | | - | | 98 (3 RCTs) | | ⨁⨁◯◯ LOW ^a,b^ |  |
| PI% reduction (3 months) | | The mean PI% reduction (3 months) was **17.23** % | | MD **5 % higher** (0.8 higher to 9.21 higher) | | - | | 265 (6 RCTs) | | ⨁⨁◯◯ LOW ^a,b^ |  |
| PI% reduction (6 months) | | The mean PI% reduction (6 months) was **25.39** mm | | MD **1.84 mm higher** (3.97 lower to 7.64 higher) | | - | | 164 (4 RCTs) | | ⨁◯◯◯ VERY LOW ^a,b,c^ |  |
| ***The risk in the intervention group** (and its 95% confidence interval) is based on the assumed risk in the comparison group and the **relative effect** of the intervention (and its 95% CI).   **CI:** Confidence interval; **MD:** Mean difference | | | | | | | | | | | |
| **GRADE Working Group grades of evidence** **High certainty:** We are very confident that the true effect lies close to that of the estimate of the effect **Moderate certainty:** We are moderately confident in the effect estimate: The true effect is likely to be close to the estimate of the effect, but there is a possibility that it is substantially different **Low certainty:** Our confidence in the effect estimate is limited: The true effect may be substantially different from the estimate of the effect **Very low certainty:** We have very little confidence in the effect estimate: The true effect is likely to be substantially different from the estimate of effect | | | | | | | | | | | |

#### Explanations

a. Downgraded due to heterogeneity

b. Downgraded due to differences in HM dosage / characteristics

c. Downgraded due to 95% CI boundaries

| **Summary of findings:** | | | | | | |
| --- | --- | --- | --- | --- | --- | --- |
| **NSPT + Probiotics compared to NSPT (+ Placebo) [4 - 6 mm] for periodontitis** | | | | | | |
| **Patient or population**: periodontitis  **Setting**:  **Intervention**: NSPT + Probiotics  **Comparison**: NSPT (+ Placebo) [4 - 6 mm] | | | | | | |
| Outcomes | **Anticipated absolute effects^*^** (95% CI) | | Relative effect (95% CI) | № of participants  (studies) | Certainty of the evidence (GRADE) | Comments |
|  | **Risk with NSPT (+ Placebo) [4 - 6 mm]** | **Risk with NSPT + Probiotics** |  |  |  |  |
| PD reduction (3 months) | The mean PD reduction (3 months) was **1.38** mm | MD **0.15 mm higher** (0.02 higher to 0.28 higher) | - | 119 (3 RCTs) | ⨁⨁⨁◯ MODERATE ^a^ |  |
| CAL gain (3 months) | The mean CAL gain (3 months) was **0.89** mm | MD **0.27 mm higher** (0.05 higher to 0.49 higher) | - | 119 (3 RCTs) | ⨁⨁◯◯ LOW ^a,b^ |  |
| ***The risk in the intervention group** (and its 95% confidence interval) is based on the assumed risk in the comparison group and the **relative effect** of the intervention (and its 95% CI).   **CI:** Confidence interval; **MD:** Mean difference | | | | | | |
| **GRADE Working Group grades of evidence** **High certainty:** We are very confident that the true effect lies close to that of the estimate of the effect **Moderate certainty:** We are moderately confident in the effect estimate: The true effect is likely to be close to the estimate of the effect, but there is a possibility that it is substantially different **Low certainty:** Our confidence in the effect estimate is limited: The true effect may be substantially different from the estimate of the effect **Very low certainty:** We have very little confidence in the effect estimate: The true effect is likely to be substantially different from the estimate of effect | | | | | | |

#### Explanations

a. Downgraded due to differences in HM dosage / characteristics

b. Downgraded due to heterogeneity

| **Summary of findings:** | | | | | | |
| --- | --- | --- | --- | --- | --- | --- |
| **NSPT + Probiotics compared to NSPT (+ Placebo) [>= 7 mm] for periodontitis** | | | | | | |
| **Patient or population**: periodontitis  **Setting**:  **Intervention**: NSPT + Probiotics  **Comparison**: NSPT (+ Placebo) [>= 7 mm] | | | | | | |
| Outcomes | **Anticipated absolute effects^*^** (95% CI) | | Relative effect (95% CI) | № of participants  (studies) | Certainty of the evidence (GRADE) | Comments |
|  | **Risk with NSPT (+ Placebo) [>= 7 mm]** | **Risk with NSPT + Probiotics** |  |  |  |  |
| PD reduction (3 months) | The mean PD reduction (3 months) was **2.30** mm | MD **0.49 mm higher** (0.03 higher to 0.96 higher) | - | 147 (4 RCTs) | ⨁⨁◯◯ LOW ^a,b^ |  |
| CAL gain (3 months) | The mean CAL gain (3 months) was **1.20** mm | MD **0.66 mm higher** (0.08 lower to 1.39 higher) | - | 147 (4 RCTs) | ⨁◯◯◯ VERY LOW ^a,b,c^ |  |
| ***The risk in the intervention group** (and its 95% confidence interval) is based on the assumed risk in the comparison group and the **relative effect** of the intervention (and its 95% CI).   **CI:** Confidence interval; **MD:** Mean difference | | | | | | |
| **GRADE Working Group grades of evidence** **High certainty:** We are very confident that the true effect lies close to that of the estimate of the effect **Moderate certainty:** We are moderately confident in the effect estimate: The true effect is likely to be close to the estimate of the effect, but there is a possibility that it is substantially different **Low certainty:** Our confidence in the effect estimate is limited: The true effect may be substantially different from the estimate of the effect **Very low certainty:** We have very little confidence in the effect estimate: The true effect is likely to be substantially different from the estimate of effect | | | | | | |

#### Explanations

a. Downgraded due to heterogeneity

b. Downgraded due to differences in HM dosage / characteristics

c. Downgraded due to 95% CI boundaries

| **Summary of findings:** | | | | | | |
| --- | --- | --- | --- | --- | --- | --- |
| **NSPT + *L. reuteri* compared to NSPT (+ Placebo) for periodontitis** | | | | | | |
| **Patient or population**: periodontitis  **Setting**:  **Intervention**: NSPT + *L. reuteri*  **Comparison**: NSPT (+ Placebo) | | | | | | |
| Outcomes | **Anticipated absolute effects^*^** (95% CI) | | Relative effect (95% CI) | № of participants  (studies) | Certainty of the evidence (GRADE) | Comments |
|  | **Risk with NSPT (+ Placebo)** | **Risk with NSPT + *L. reuteri*** |  |  |  |  |
| PD reduction (3 months) | The mean PD reduction (3 months) was **1.29** mm | mean **0.33 mm higher** (0.08 higher to 0.58 higher) | - | 233 (6 RCTs) | ⨁⨁⨁◯ MODERATE ^a^ |  |
| PD reduction (6 months) | The mean PD reduction (6 months) was **1.44** mm | **0.41 mm higher** (0.37 lower to 1.19 higher) | - | 175 (4 RCTs) | ⨁⨁◯◯ LOW ^a,b^ |  |
| CAL gain (3 months) | The mean CAL gain (3 months) was **0.38** mm | mean **0.26 mm higher** (0.14 higher to 0.38 higher) | - | 233 (6 RCTs) | ⨁⨁⨁◯ MODERATE ^a^ |  |
| CAL gain (6 months) | The mean CAL gain (6 months) was **0.38** mm | mean **0.43 mm higher** (0.07 lower to 0.92 higher) | - | 175 (4 RCTs) | ⨁⨁◯◯ LOW ^a,b^ |  |
| BOP% reduction (3 months) | The mean BOP% reduction (3 months) was **43.17** % | mean **5.44 % higher** (0.31 higher to 10.56 higher) | - | 233 (6 RCTs) | ⨁⨁◯◯ LOW ^a,b^ |  |
| BOP% reduction (6 months) | The mean BOP% reduction (6 months) was **49.28** % | mean **2.24 % higher** (4.18 lower to 8.65 higher) | - | 175 (4 RCTs) | ⨁⨁◯◯ LOW ^a,b^ |  |
| ***The risk in the intervention group** (and its 95% confidence interval) is based on the assumed risk in the comparison group and the **relative effect** of the intervention (and its 95% CI).   **CI:** Confidence interval | | | | | | |
| **GRADE Working Group grades of evidence** **High certainty:** We are very confident that the true effect lies close to that of the estimate of the effect **Moderate certainty:** We are moderately confident in the effect estimate: The true effect is likely to be close to the estimate of the effect, but there is a possibility that it is substantially different **Low certainty:** Our confidence in the effect estimate is limited: The true effect may be substantially different from the estimate of the effect **Very low certainty:** We have very little confidence in the effect estimate: The true effect is likely to be substantially different from the estimate of effect | | | | | | |

Explanations

a. Downgraded due to heterogeneity

b. Downgraded due to 95% Ci boundaries

| **Summary of findings:** | | | | | | |
| --- | --- | --- | --- | --- | --- | --- |
| **NSPT + Melatonin compared to NSPT (+ Placebo) for periodontitis** | | | | | | |
| **Patient or population**: periodontitis  **Setting**:  **Intervention**: NSPT + Melatonin  **Comparison**: NSPT (+ Placebo) | | | | | | |
| Outcomes | **Anticipated absolute effects^*^** (95% CI) | | Relative effect (95% CI) | № of participants  (studies) | Certainty of the evidence (GRADE) | Comments |
|  | **Risk with NSPT (+ Placebo)** | **Risk with NSPT + Melatonin** |  |  |  |  |
| PD reduction (6 months) | The mean PD reduction (6 months) was **1.27** mm | MD **0.85 mm higher** (0.46 higher to 1.24 higher) | - | 134 (3 RCTs) | ⨁◯◯◯ VERY LOW ^a,b,c^ |  |
| ***The risk in the intervention group** (and its 95% confidence interval) is based on the assumed risk in the comparison group and the **relative effect** of the intervention (and its 95% CI).   **CI:** Confidence interval; **MD:** Mean difference | | | | | | |
| **GRADE Working Group grades of evidence** **High certainty:** We are very confident that the true effect lies close to that of the estimate of the effect **Moderate certainty:** We are moderately confident in the effect estimate: The true effect is likely to be close to the estimate of the effect, but there is a possibility that it is substantially different **Low certainty:** Our confidence in the effect estimate is limited: The true effect may be substantially different from the estimate of the effect **Very low certainty:** We have very little confidence in the effect estimate: The true effect is likely to be substantially different from the estimate of effect | | | | | | |

#### Explanations

a. Downgraded due to risk of bias

b. Downgraded due to heterogeneity

c. Downgraded due to differences in dosages

| **Summary of findings:** | | | | | | |
| --- | --- | --- | --- | --- | --- | --- |
| **NSPT + Sub Tetracycline compared to NSPT (+ Placebo) for periodontitis** | | | | | | |
| **Patient or population**: periodontitis  **Setting**:  **Intervention**: NSPT + Sub Tetracycline  **Comparison**: NSPT (+ Placebo) | | | | | | |
| Outcomes | **Anticipated absolute effects^*^** (95% CI) | | Relative effect (95% CI) | № of participants  (studies) | Certainty of the evidence (GRADE) | Comments |
|  | **Risk with NSPT (+ Placebo)** | **Risk with NSPT + Sub Tetracycline** |  |  |  |  |
| PD reduction (3 months) | The mean PD reduction (3 months) - All sites was **1.85** mm | MD **0.2 mm higher** (0 to 0.4 higher) | - | 449 (5 RCTs) | ⨁◯◯◯ VERY LOW ^a,b,c^ |  |
| CAL gain (3 months) | The mean CAL gain (3 months) - All sites was **0.99** mm | MD **0.3 mm higher** (0.19 higher to 0.41 higher) | - | 327 (4 RCTs) | ⨁⨁◯◯ LOW ^a,b^ |  |
| ***The risk in the intervention group** (and its 95% confidence interval) is based on the assumed risk in the comparison group and the **relative effect** of the intervention (and its 95% CI).   **CI:** Confidence interval; **MD:** Mean difference | | | | | | |
| **GRADE Working Group grades of evidence** **High certainty:** We are very confident that the true effect lies close to that of the estimate of the effect **Moderate certainty:** We are moderately confident in the effect estimate: The true effect is likely to be close to the estimate of the effect, but there is a possibility that it is substantially different **Low certainty:** Our confidence in the effect estimate is limited: The true effect may be substantially different from the estimate of the effect **Very low certainty:** We have very little confidence in the effect estimate: The true effect is likely to be substantially different from the estimate of effect | | | | | | |

#### Explanations

a. Downgraded due to risk of bias

b. Downgraded due to heterogeneity

c. Downgraded due to 95% CI boundaries

***Appendix 6***

Abbreviations used in Table 5

AIS = Athens insomnia scale

API = Approximal plaque index

BI = bleeding index

BMD = bone mineral density

BOP = bleeding on probing

BSAP = bone-specific alkaline phosphatase

CAL = clinical attachment level

CFUs = colony-forming units

CHX = chlorhexidine

CP = chronic periodontitis

CRP = C-reactive protein

DHA = docosahexaenoic acid

EPA = eicosapentaenoic acid

F = female

FSH = Follicle-stimulating hormone

FV = fruit + vegetables antioxidant juice powder phytonutrient

FVB = Fruit + vegetables + berries antioxidant juice powder phytonutrient

GBI = gingival bleeding index

GCF = gingival crevicular fluid

GI = gingival index

IL-1β = interleukin 1b

LH = luteinizing hormone

M = male

MCP-3 = monocyte chemoattractant protein-3

MGI = modified gingival index

MMP = matrix metalloproteinase

Mo = months

ND = not described

NOS = Nitric Oxide Synthase

NSPT = non-surgical periodontal therapy

NTx = cross-linked N-telopeptide

OHI = oral hygienic instructions

OHIS = oral hygiene index-simplified

PD = probing depth

PI = plaque index

PUFAs = polyunsaturated fatty acids

RAL = relative attachment levels

RANKL = receptor activator of nuclear factor-kappa B ligand

REC = recession

SBI = sulcus bleeding index

SD = standard deviation

SOD = superoxide dismutase

SRP = scaling and root planing

t-PA = tissue plasminogen activator

T2DM = type-2 diabetes mellitus

TAOC = total antioxidant capacity

TIMP = tissue inhibitor of metalloproteinases

TNF-α = tumor necrosis factor- α

VAS = visual analogue scale

References

[1] Ainamo, Jukka, and I. Bay. 1975. Problems and proposals for recording gingivitis and plaque. International Dental Journal 25: 229-235.

[2] Armitage, Gary C. 1999. Development of a classification system for periodontal diseases and conditions. Annals of Periodontology 4: 1-6. 10.1902/annals.1999.4.1.1

[3] Caton, Jack G., Gary C. Armitage, Tord Berglundh, Iain L. C. Chapple, Søren Jepsen, Kenneth S. Kornman, et al. 2018. A new classification scheme for periodontal and peri-implant diseases and conditions - Introduction and key changes from the 1999 classification. Journal of Periodontology 89 Suppl 1: S1-S8. 10.1002/JPER.18-0157

[4] Flemmig, Thomas F. 1999. Periodontitis. Annals of Periodontology 4: 32-38. 10.1902/annals.1999.4.1.32

[5] Gordon, J. M., I. B. Lamster, and M. C. Seiger. 1985. Efficacy of Listerine antiseptic in inhibiting the development of plaque and gingivitis. Journal of Clinical Periodontology 12: 697-704. 10.1111/j.1600-051x.1985.tb00941.x

[6] Lange, D. E., H. C. Plagmann, A. Eenboom, and A. Promesberger. 1977. Clinical methods for the objective evaluation of oral hygiene. Deutsche Zahnarztliche Zeitschrift 32: 44-47.

[7] Lobene, Ralph R., Pramod M. Soparkar, and M. B. Newman. 1982. Use of dental floss. Effect on plaque and gingivitis. Clinical Preventive Dentistry 4: 5-8.

[8] Lobene, Ralph R., T. Weatherford, N. M. Ross, R. A. Lamm, and L. Menaker. 1986. A modified gingival index for use in clinical trials. Clinical Preventive Dentistry 8: 3-6.

[9] Loe, H., and J. Silness. 1963. Periodontal Disease in Pregnancy. I. Prevalence and Severity. Acta Odontol Scand 21: 533-551. 10.3109/00016356309011240

[10] Machtei, Eli E., Larss A. Christersson, Sara G. Grossi, Robert Dunford, Joseph J. Zambon, and Robert J. Genco. 1992. Clinical criteria for the definition of "established periodontitis". Journal of Periodontology 63: 206-214. 10.1902/jop.1992.63.3.206

[11] O'leary, Timothy J., Robert B. Drake, and James E. Naylor. 1972. The plaque control record. Journal of Periodontology 43: 38. 10.1902/jop.1972.43.1.38

[12] Page, Roy C., and Paul I. Eke. 2007. Case Definitions for Use in Population-Based Surveillance of Periodontitis. Journal of Periodontology 78 Suppl 7S: 1387-1399. 10.1902/jop.2007.060264

[13] Periodontology, American Academy Of. 2000. Parameter on chronic periodontitis with slight to moderate loss of periodontal support. Journal of Periodontology 71: 853-855. 10.1902/jop.2000.71.5-S.853

[14] Polson, A. M., G. L. Southard, R. L. Dunn, A. P. Polson, J. R. Billen, and L. L. Laster. 1995. Initial study of guided tissue regeneration in Class II furcation defects after use of a biodegradable barrier. International Journal of Periodontics and Restorative Dentistry 15: 42-55.

[15] Pozo, P., M. A. Valenzuela, C. Melej, M. Zaldivar, J. Puente, B. Martinez, and J. Gamonal. 2005. Longitudinal analysis of metalloproteinases, tissue inhibitors of metalloproteinases and clinical parameters in gingival crevicular fluid from periodontitis-affected patients. Journal of Periodontal Research 40: 199-207. 10.1111/j.1600-0765.2005.00786.x

[16] Quirynen, Marc, Marc De Soete, Geert Boschmans, Martine Pauwels, Wim Coucke, Wim Teughels, and Daniel Van Steenberghe. 2006. Benefit of "one-stage full-mouth disinfection" is explained by disinfection and root planing within 24 hours: a randomized controlled trial. Journal of Clinical Periodontology 33: 639-647. 10.1111/j.1600-051X.2006.00959.x

[17] Quirynen, Marc, Claudio Mongardini, Marc De Soete, Martine Pauwels, Wim Coucke, Johan Van Eldere, and Daniel Van Steenberghe. 2000. The role of chlorhexidine in the one-stage full-mouth disinfection treatment of patients with advanced adult periodontitis. Long-term clinical and microbiological observations. Journal of Clinical Periodontology 27: 578-589. 10.1034/j.1600-051x.2000.027008578.x

[18] Silness, J., and H. Loe. 1964. Periodontal Disease in Pregnancy. Ii. Correlation between Oral Hygiene and Periodontal Condtion. Acta Odontol Scand 22: 121-135. 10.3109/00016356408993968

[19] Tonetti, Maurizio S., Henry Greenwell, and Kenneth S. Kornman. 2018. Staging and grading of periodontitis: Framework and proposal of a new classification and case definition. Journal of Periodontology 89 Suppl 1: S159-S172. 10.1002/JPER.18-0006

[20] Tonetti, Maurizio S., Henry Greenwell, and Kenneth S. Kornman. 2018. Staging and grading of periodontitis: Framework and proposal of a new classification and case definition. Journal of Clinical Periodontology 45 Suppl 20: S149-S161. 10.1111/jcpe.12945

[21] Turesky, Samuel, Neville D. Gilmore, and Irving Glickman. 1970. Reduced plaque formation by the chloromethyl analogue of victamine C. Journal of Periodontology 41: 41-43. 10.1902/jop.1970.41.41.41

[22] Van Der Velden, Ubele. 2005. Purpose and problems of periodontal disease classification. Periodontology 2000 39: 13-21. 10.1111/j.1600-0757.2005.00127.x
